# Cardiomyocyte and stromal cell cross-talk influences the pathogenesis of arrhythmogenic cardiomyopathy: a multi-level analysis uncovers DLK1-NOTCH pathway role in fibro-adipose remodelling

**DOI:** 10.1038/s41420-024-02232-8

**Published:** 2024-11-28

**Authors:** Angela Serena Maione, Lara Iengo, Luca Sala, Ilaria Massaiu, Mattia Chiesa, Melania Lippi, Stefania Ghilardi, Chiara Florindi, Francesco Lodola, Antonio Zaza, Claudio Tondo, Marco Schiavone, Cristina Banfi, Giulio Pompilio, Paolo Poggio, Elena Sommariva

**Affiliations:** 1 Unit of Vascular Biology and Regenerative Medicine, Centro Cardiologico Monzino IRCCS, 20138 Milan, Italy; 2 Center for Cardiac Arrhythmias of Genetic Origin and Laboratory of Cardiovascular Genetics, Istituto Auxologico Italiano IRCCS, 20095 Milan, Italy; 3 Department of Biotechnology and Biosciences, University of Milano-Bicocca, Milan, 20126 Italy; 4 Unit for the Study of Aortic, Valvular and Coronary Pathologies, Centro Cardiologico Monzino IRCCS, 20138 Milan, Italy; 5 Bioinformatics and Artificial Intelligence Facility, Centro Cardiologico Monzino IRCCS, 20138 Milan, Italy; 6 Department of Electronics, Information and Biomedical Engineering, Politecnico di Milano, 20133 Milan, Italy; 7 Unit of Functional Proteomics, Metabolomics, and Network Analysis, Centro Cardiologico Monzino IRCCS, 20138 Milan, Italy; 8 Department of Clinical Electrophysiology and Cardiac Pacing, Centro Cardiologico Monzino IRCCS, 20138 Milan, Italy; 9 Department of Biomedical, Surgical and Dental Sciences, Università degli Studi di Milano, 20122 Milan, Italy; 10 Department of Systems Medicine, University of Rome Tor Vergata, 00133 Rome, Italy

**Keywords:** Heart stem cells, Cardiomyopathies

## Abstract

Arrhythmogenic Cardiomyopathy (ACM) is a life-threatening, genetically determined disease primarily caused by mutations in desmosomal genes, such as *PKP2*. Currently, there is no etiological therapy for ACM due to its complex and not fully elucidated pathogenesis. Various cardiac cell types affected by the genetic mutation, such as cardiomyocytes (CM) and cardiac mesenchymal stromal cells (cMSC), individually contribute to the ACM phenotype, driving functional abnormalities and fibro-fatty substitution, respectively. However, the relative importance of the CM and cMSC alterations, as well as their reciprocal influence in disease progression remain poorly understood. We hypothesised that ACM-dependent phenotypes are driven not only by alterations in individual cell types but also by the reciprocal interactions between CM and cMSC, which may further impact disease pathogenesis. We utilized a patient-specific, multicellular cardiac system composed of either control or *PKP2*-mutated CM and cMSC to assess the mutation’s role in fibro-fatty phenotype by immunofluorescence, and contractile behaviour of co-cultures using cell motion detection software. Additionally, we investigated reciprocal interactions both in silico and via multi-targeted proteomics. We demonstrated that ACM CM can promote fibro-adipose differentiation of cMSC. Conversely, ACM cMSC contribute to increasing the rate of abnormal contractile events with likely arrhythmic significance. Furthermore, we showed that an ACM-causative mutation alters the CM-cMSC interaction pattern. We identified the CM-sourced DLK1 as a novel regulator of fibro-adipose remodelling in ACM. Our study challenges the paradigm of exclusive cell-specific mechanisms in ACM. A deeper understanding of the cell-cell influence is crucial for identifying novel therapeutic targets for ACM, and this concept is exploitable for other cardiomyopathies.

## Introduction

The heart is composed of different cell types that interact closely to maintain cardiac homeostasis. Although cardiomyocytes (CM) occupy the largest volume, other cell types are numerically predominant [1–3]. This suggests the presence of cell-specific functions and numerous interactions that coordinate cellular activity and support their functional cooperation, thereby regulating overall heart function. These complex interactions occur both under physiological conditions and during disease development [4].

Arrhythmogenic Cardiomyopathy (ACM) is a rare genetic heart condition that affects predominantly young adults. In ACM, the cardiac tissue is pathologically remodelled, and ventricular arrhythmias are the major causes of sudden death [5].

Historically, CM have been the primary focus of ACM pathogenesis studies as they are the main determinants of electrical and mechanical cardiac activities, which are impaired in ACM [6]. ACM is primarily associated with mutations in desmosomal genes such as *PKP2*, which encodes for Plakophilin-2 [7]. Mechanical continuity, provided by desmosomes, is preferentially located at CM intercalated discs. In addition to impaired ventricular systolic function, ACM is characterized by fibro-adipose replacement of the myocardium. Among the different hypotheses regarding its origin, most agree that this replacement is sustained by enhanced differentiation of cardiac Mesenchymal Stromal Cells (cMSC) [8–13]. Under normal physiological conditions, cMSC support the structural and functional integrity of the myocardium [14]. It has been shown that these cells express desmosomal genes [8], and therefore they are subject to the desmosomal mutation effects, being key determinants of ACM pathogenesis [8]. Previous data from our group have highlighted that ACM cMSC mediate the fibro-adipose accumulation process based on their commitment to differentiate into myofibroblasts and adipocytes [8, 9].

Despite the presence of dead or impaired CM and the abundance of neighbouring cMSC genetically predisposed to aberrant differentiation in the ACM heart, an in-depth study clarifying how these cells reciprocally affect each other during ACM progression is lacking. Here, we hypothesise that the reciprocal interaction between CM and cMSC may have an additional impact on disease pathogenesis, beyond the individual cell type contribution. To address this, we generated a patient-specific co-culture model combining induced pluripotent stem cells derived CM (hiPSC-CM) and primary cMSC. The ability to replace each healthy cell type (CM or cMSC) with a counterpart carrying an ACM genotype allowed us to assess the specific contribution of each cell population to the alterations typical of the disease. We chose to use, as representative of the ACM genotype, primary cells and hiPSC from a carrier of a *PKP2* mutation, the most frequently mutated gene in ACM patients, with a prevalence ranging between 10 and 45% [15, 16]. Specifically, we: (i) provided evidence that ACM CM can influence the fibro-adipose commitment of cMSC; (ii) validated that ACM cMSC can affect CM contraction by increasing the incidence of events of arrhythmic significance; (iii) demonstrated that the ACM-causative mutation results in variations in the interaction pattern between CM and cMSC; (iv) confirmed, through a secretome profiling, that CM-cMSC interplay and regulation in ACM also occur by paracrine signalling; and (v) identified a novel CM-derived factor that regulates cMSC fibro-adipose differentiation.

Given the crucial role of interactions between cMSC and CM in maintaining cardiac tissue integrity, defining the heterotypic cellular interaction network in ACM is of critical translational importance. Indeed, elucidating the cellular pathways triggered by direct and paracrine interactions could represent a valuable tool for identifying potential pharmacological targets.

## Results

### ACM cardiomyocytes promote fibro-adipose differentiation ability of mesenchymal stromal cells

**Fig. 1:**
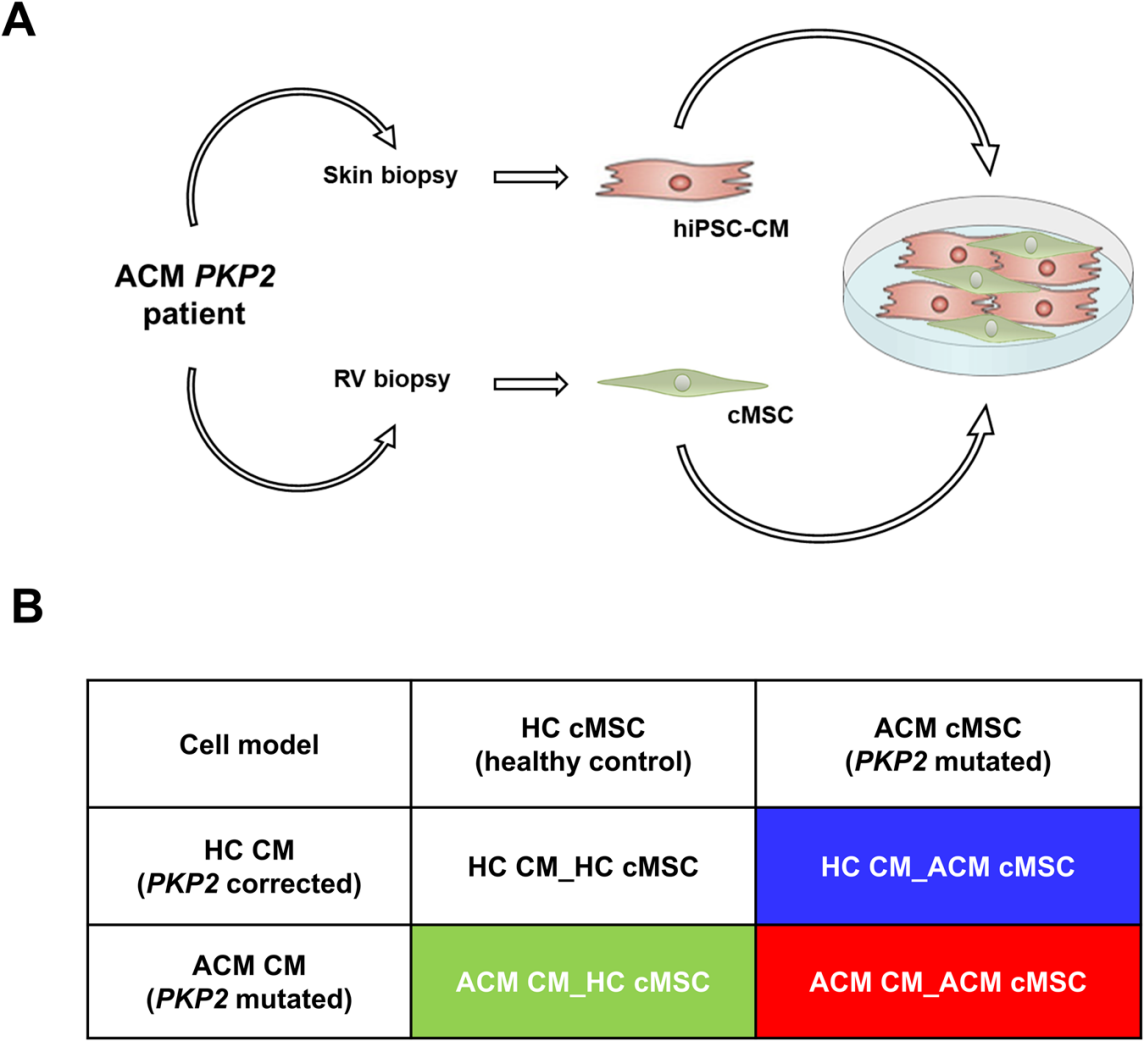
Strategy for the generation of co-cultures. **A** Primary stromal cells carrying the *PKP2* mutation (c.2013delC) were collected from a right ventricle biopsy sample, and sex/aged-matched control stromal cells were selected. iPSC reprogrammed from the same ACM patient and from an isogenic line with the *PKP2* mutation were used. Cardiac co-cultures were assembled by combining 85% iPSC-CM and 15% primary cMSC. **B** Four different co-culture models have been produced by combining iPSC-CM and cMSC, either mutated or control, in all possible combinations (1) Healthy control cardiomyocytes and healthy control stromal cells (HC CM_HC cMSC) in white; (2) Healthy control cardiomyocytes and Arrhythmogenic Cardiomyopathy (ACM) stromal cells (HC CM_ACM cMSC) in blue; (3) ACM cardiomyocytes and healthy control stromal cells (ACM CM_HC cMSC) in green; (4) ACM cardiomyocytes and ACM stromal cells (ACM CM_ACM cMSC) in red. The abbreviations used to denote the four co-cultures (and their corresponding colours) are repeated in the following figures.

ACM hearts undergo fibro-adipose remodelling, primarily due to the alteration of cMSC and their increased propensity to differentiate into adipocytes and myofibroblasts [8, 9, 17]. To verify whether CM contribute to cMSC fibro-adipogenic differentiation, four different co-cultures were assembled by combining control (HC) or mutated (ACM) hiPSC-CM with primary cMSC, as described in Fig. 1 and detailed in the methods section. The co-cultures were maintained in BPEL for ten days, after which immunofluorescence and fluorescent staining analyses of Collagen I and Nile Red were performed to evaluate the fibrotic and adipose accumulation, respectively.

**Fig. 2:**
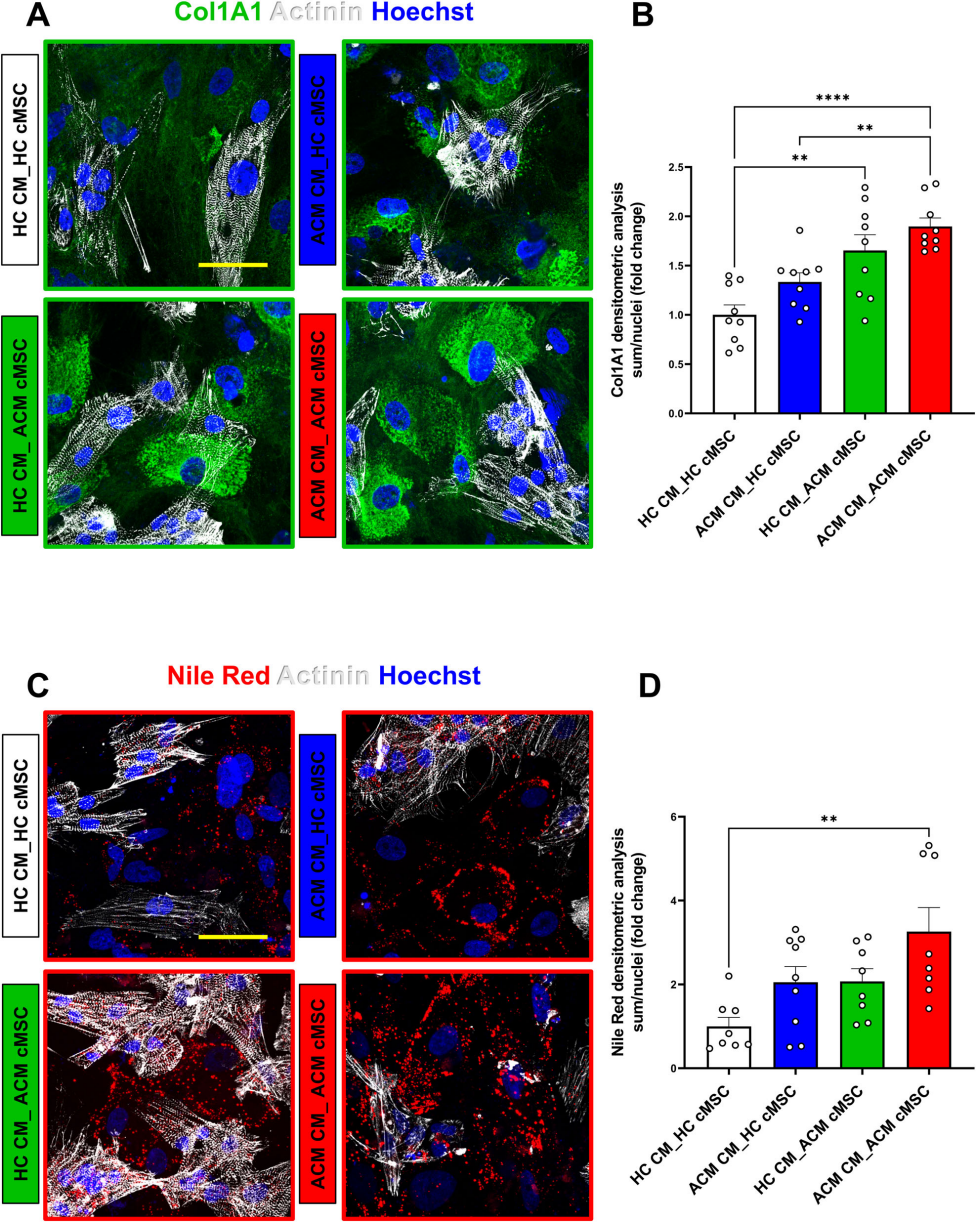
Fibro-adipose accumulation in co-culture models. **A**, **C** Representative images of immunostaining for Col1A1 (green), Nile Red (red), and alpha-Actinin (white), markers of myofibroblasts, adipocytes and cardiomyocytes respectively. Nuclei are stained with Hoechst 33342 (blue). Magnification: 40X; scale bar: 50 μm. **B**, **D** Quantification of the images shown as fold change with respect to HC co-culture model (HC CM_HC cMSC) with *p*-values from One-way ANOVA and Tukey’s post-test are indicated in each panel. For Col1A1 staining: n = 9 for HC CM_HC MSC; n = 9 for ACM CM_ACM MSC; n = 9 for ACM CM_HC MSC; n = 9 for HC CM_ACM MSC. For Nile Red staining n = 8 for HC CM_HC MSC; n = 9 for ACM CM_ACM MSC; n = 8 for ACM CM_HC MSC; n = 8. Three independent differentiations were used for both Col1A1 and Nile Red staining. Data information: mean ± SEM. **P < 0.01 ****P ≤ 0.0001.

The analysis revealed that the production of Collagen I was higher in ACM co-culture (ACM CM_ACM cMSC) compared to the HC (HC CM_HC cMSC) co-culture (Fig. 2A, B). Intermediate levels of Collagen I were observed in the mixed co-cultures (ACM CM_HC cMSC and HC CM_ACM cMSC) (Fig. 2A, B). Similarly, Nile Red staining revealed that the ACM co-culture (ACM CM_ACM cMSC) accumulated lipid droplets to a greater extent than the HC (HC CM_HC cMSC) co-culture (Fig. 2C, D). Intermediate and comparable levels of lipid accumulation were observed in the mixed co-cultures (ACM CM_HC cMSC and HC CM_ACM cMSC) (Fig. 2C, D).

These data collectively demonstrate that the presence of ACM CM in co-culture promotes the accumulation of lipids and collagen by HC cMSC, indicating that CM can influence stromal-dependent fibro-adipose remodelling.

### ACM stromal cells contribute to cardiomyocyte contractile dysfunction and arrhythmic susceptibility

**Fig. 3:**
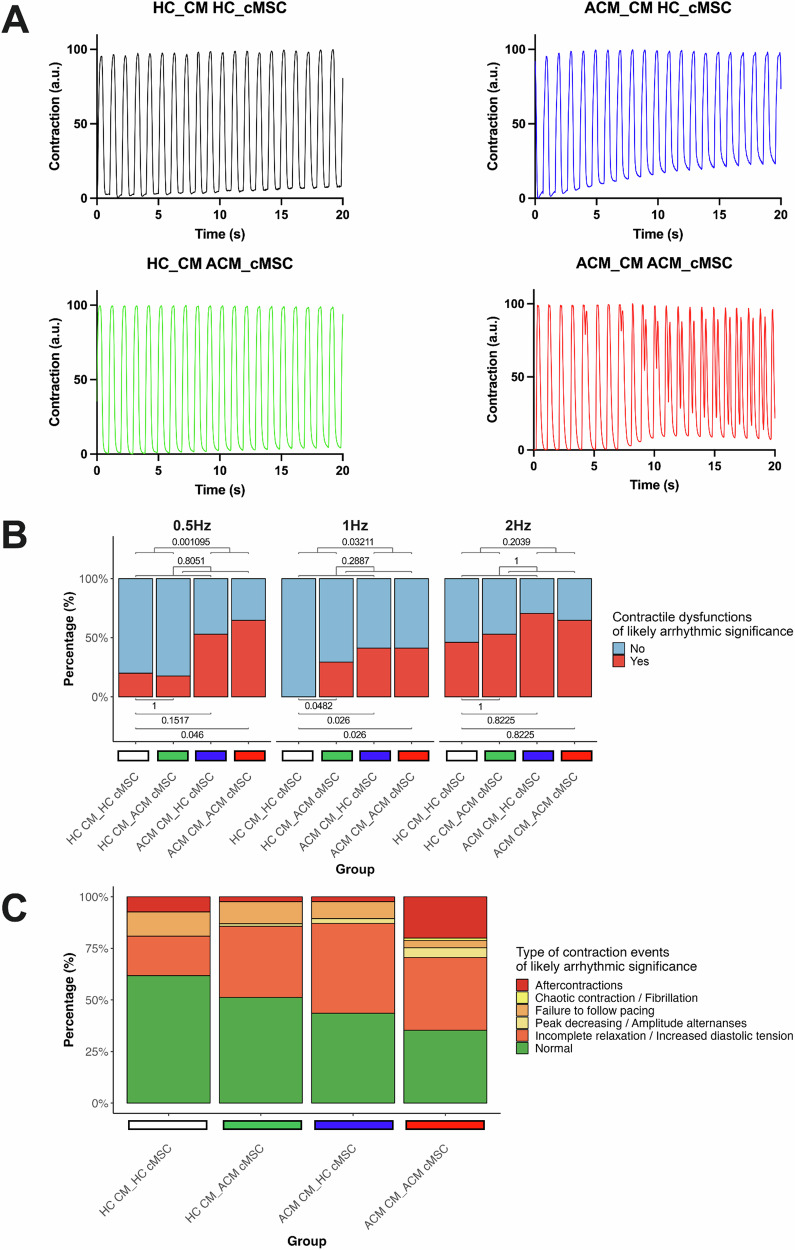
Occurrence of contractile anomalies of arrhythmic significance in co-cultures. **A** Representative contraction profiles obtained with MUSCLEMOTION from co-cultured monolayers paced at 1 Hz, measured in arbitrary units (a.u.). Proarrhythmic events such as baseline drift (blue), decreasing peak (green) or aftercontraction (red) are visible. **B** Proportion of monolayers exhibiting normal (blue) or likely arrhythmic (red) contractile events at different pacing frequencies. *P*-values from Fisher’s exact tests and the respective pairwise comparisons are indicated. **C** Combined proportion and distribution of contractile abnormalities with potential relevance to arrhythmogenesis across the four co-culture conditions at all pacing frequencies. Data were generated from three independent batches of differentiation. N = 15 for HC CM_HC MSC; n = 17 for ACM CM_ACM MSC; n = 17 for ACM CM_HC MSC; n = 17 for HC CM_ACM MSC. Three independent differentiations were used.

To assess the influence of each cell type on contractile function, high-speed movies of the four different co-cultures were analysed using MUSCLEMOTION [18] to extract the contractile patterns at various pacing rates. An increase in the occurrence of contractile anomalies, likely of arrhythmic significance, was observed when transitioning from purely HC genotypes to purely ACM genotypes. The inclusion of ACM CM in co-cultured monolayers significantly increased the percentage of these events compared to co-cultures containing HC CM; particularly at pacing rates of 0.5 Hz (Fisher’s exact test, *p* = 0.001095) and 1 Hz (Fisher’s exact test, *p* = 0.03211), while no significant changes observed at 2 Hz (Fig. 3A, B; Supplementary Fig. S2; Supplementary File Video S1–4, Fisher’s exact test, *p* = 0.2039).

Overall, the inclusion of ACM cMSC in co-cultured monolayers did not increase the percentage of contractile anomalies of likely arrhythmic significance compared to co-cultures with HC cMSC at any frequency (Fisher’s exact test, *p* = 0.8051 at 0.5 Hz, *p* = 0.2887 at 1 Hz, *p* = 1 at 2 Hz). However, the presence of ACM cMSC alone in the co-culture (HC CM_ACM cMSC) increased the percentage of contractile dysfunctions of likely arrhythmic significance at 1 Hz (Fig. 3A, B; Supplementary Fig. S2; Supplementary File Video S1–4, Fisher’s exact test, *p* = 0.0482) but not at 0.5 Hz or 2 Hz (Fisher’s exact test, *p* = 1) when compared to the HC CM_HC cMSC model.

Qualitative stratification of proarrhythmic events by type (Fig. 3C; Supplementary File Video S1–4) revealed a progressive decrease in the fraction of monolayers exhibiting a normal contractile phenotype, with a higher incidence of monolayers displaying an upward drift at baseline (i.e., incomplete and defective relaxation), aftercontractions, or monolayers that escaped the imposed pacing frequency (a representation of the observed abnormal contractile phenotype is shown in Supplementary Fig. S1A–F). Quantitative analyses of temporal contractile parameters were performed only on co-cultures exhibiting normal contractile patterns (Supplementary Fig. S3C, D).

Overall, these results suggest that both cell types contribute to the pro-arrhythmic phenotype observed in ACM.

### Cardiomyocyte and stromal cell communication depends on their ACM / HC genotype

Our study aimed to highlight cell-cell communication dynamics by uncovering the reciprocal influence between CM and cMSC. Specifically, we sought to identify how ACM CM could potentially influence healthy stromal cells and how the ACM stromal cells might pathogenetically impact healthy cardiomyocytes. To achieve this, we performed an in-silico cell-cell communication analysis, leveraging the RNA-Seq data to detect relevant ligand-receptor pairs.

We performed RNA-seq on 12 samples (3 ACM CM, 3 ACM cMSC, 3 HC CM, and 3 HC cMSC). Out of 21,071 expressed genes, 7,864 were found to be highly differentially expressed (ANOVA adj.p-value < 0.01) in at least one of the following comparisons: 1) ACM CM vs. HC CM (2,611 genes); 2) ACM cMSC vs. HC cMSC (5,253 genes) (Supplementary Fig. S4A).

**Fig. 4:**
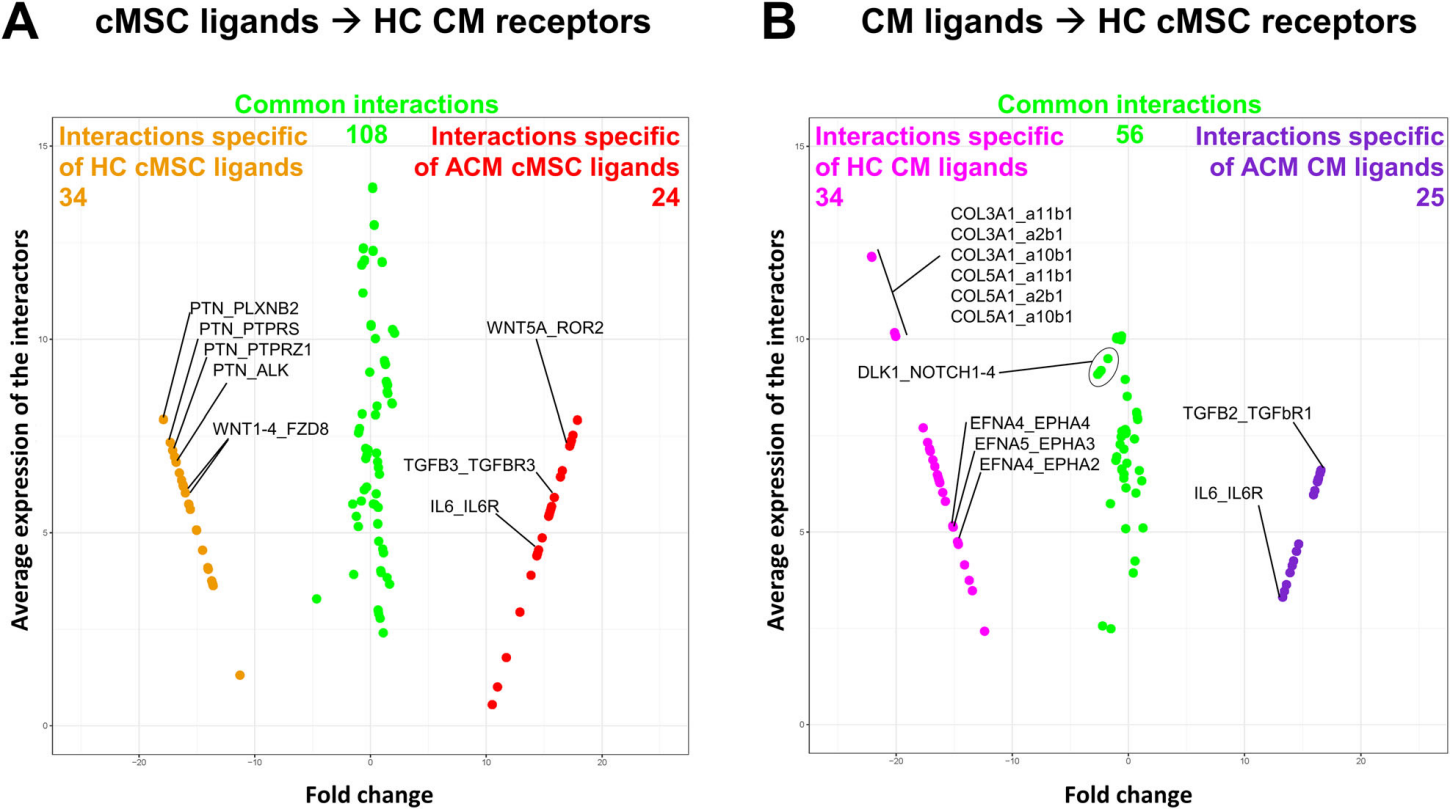
In silico-predicted interactions in co-cultures. Output of the CellphoneDB analysis of the transcriptomes from four homogeneous cell cultures (ACM CM, HC CM, ACM cMSC and HC cMSC) for in silico identification of ligand-receptor interaction. Each MA plot represents the X axis as the fold change of the interaction score when the ligand is ACM vs. HC (log_2_ fold-change of the interaction score), and on the Y axis as the average expression of the interactors (log_2_ interaction score). Each dot is a specific ligand-receptor interaction. Red and orange dots represent interactions exclusively found in cMSC ACM → CM HC and cMSC HC → CM HC (**A**), respectively, while purple and magenta dots represent interaction exclusively found in CM ACM → cMSC HC and CM HC → cMSC HC (**B**), respectively. Green dots represent the interactions that are common to the different genotypes. Only predicted interactions with *p* < 0.001 are reported. Labels, statistics and quantifications are reported in Supplementary Data S1, S2 and Supplementary Figs. S5, S6.

The entire transcriptomes were then used to identify potential crosstalk between CM and cMSC with CellPhoneDB. We predicted interactions between ligands and receptors (ligand_receptor) for each pair of cell types based on the expression of a receptor by one cell type and a ligand by the other. Each ligand_receptor pairs received an interaction score based on the total number of predicted interactions. The enrichment of these interactions for specific comparisons, depending on the genotype, is depicted in Fig. 4.

Among the 1,630 ligand-receptor pairs annotated in the CellPhoneDB database, 142, 132, 90 and 81 were found to be significantly interacting (|log_2_(FC)| > 0.5 and *p* < 0.01) when considering the interactions between HC cMSC vs HC CM (Fig. 4A, orange and green dots; Supplementary File Data S1), ACM cMSC vs. HC CM (Fig. 4A, red and green dots; File Data S1), HC CM vs. HC cMSC (Fig. 4B, magenta and green dots; Supplementary File Data S2), ACM CM vs. HC cMSC (Fig. 4B, purple and green dots; Supplementary File Data S2), respectively.

In evaluating the interaction of HC or ACM cMSC ligands with healthy CM receptors, we identified 108 common interactions, of which 72 exhibited differential score patterns. Additionally, HC cMSC had 34 distinct interactions that were absent in ACM cMSC (indicated by orange dots in Fig. 4A and Supplementary Fig. S5), whereas ACM cMSC showed 24 unique interactions with healthy CM (indicated by red dots in Fig. 4A; Supplementary Fig. S5). For example, high score interactions specific to healthy cMSC included PTN_PLXNB2 or other receptors and WNT1-4_FZD8. In contrast, IL6_IL6R, TGFB3_TGFBR3, and WNT5A_ROR2 interactions were specifically present when the cMSC were ACM (Supplementary File Data S1; Supplementary Fig. S5).

Our analysis revealed 56 interactions common to both HC and ACM CM ligands with healthy cMSC receptors. However, 20 of these interactions exhibited differential score patterns. For example, the interaction between DLK1 CM ligand and the NOTCH1-2-3-4 (DLK1_NOTCH1-2-3-4) receptors displayed lower scores when ACM CM ligands were present compared to HC CM ligands. Furthermore, HC CM ligands demonstrated 34 distinct interactions (indicated by magenta dots in Fig. 4B; Supplementary Fig. S6) that were absent when ACM CM ligands were involved, while ACM CM ligands exhibited 25 unique interactions directed towards healthy cMSC receptors (indicated by purple dots in Fig. 4B; Supplementary Fig. S6). Notably, the communication between ACM CM ligands and healthy cMSC receptors implied a loss of interactions involving the COL3A1_a2b1 and COL5A1_a11b1 complexes, and the EFNA4-5_EPHA2-3-4, compared to HC CM ligands and cMSC receptors. Conversely, ACM CM developed new interactions with healthy cMSC, such as TGFB2_TGFBR1, FGF1-18_FGFR2 and IL6_IL6R (Supplementary File Data S2; Supplementary Fig. S6).

### Cardiomyocytes and stromal cells ACM-specific regulation also occurs through paracrine signals

**Fig. 5:**
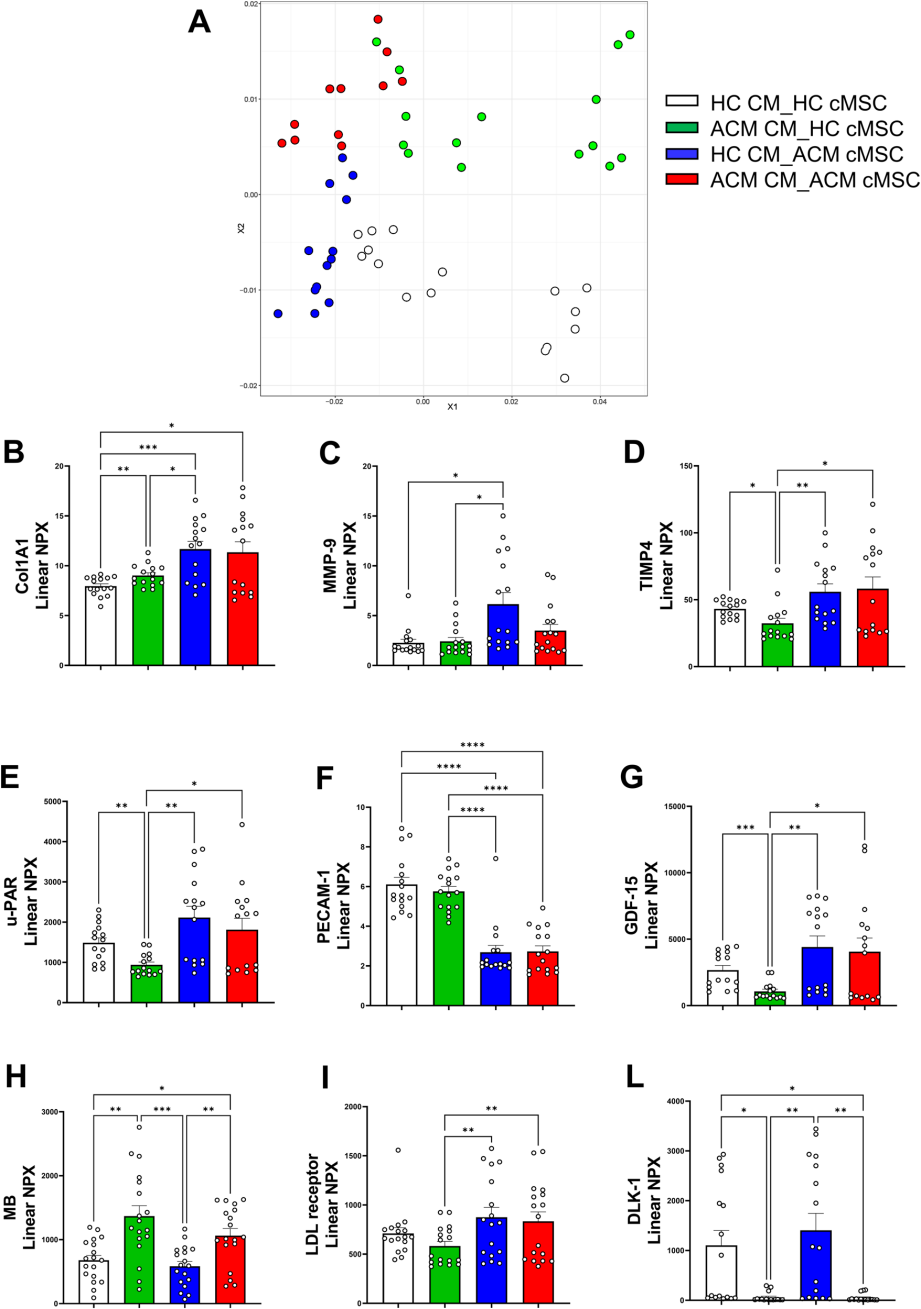
Proteins involved in paracrine interactions in co-cultures. **A** Scatterplot of the two coordinates (X1 and X2) obtained from the Multi-Dimensional Scaling (MDS) performed on 36 proteins assessed by Olink samples, representing the diversity of the secretomes from the four co-cultures. Each dot represents a sample, with colours indicating cell groups (blank: HC CM_HC cMSC; green: ACM CM_HC cMSC; blue: HC CM_ACM cMSC; red: ACM CM_ACM cMSC). Multi-Dimensional Scaling (MDS) allows representation of sample proteomes dissimilarities in a two-dimensional space. To avoid introducing noise by data imputation, only samples with complete proteomes and less than 20% of missing values were plotted. Data for all measured proteins are reported in Supplementary DATA S3. For statistical analysis of individual proteins, all samples with expression values were used. **B**–**L** Protein levels of Col1A1, MMP-9, TIMP4, uPAR, PECAM-1, MB, GDF-15, LDL-receptor and DLK-1 were measured by Olink, in conditioned media from co-cultures. Protein levels are shown as Normalized Protein eXpression (NPX) levels. P- values from One-way ANOVA and Tukey’s post-test are indicated in each panel. N = 15/18 for HC CM_HC MSC; n = 15/18 for ACM CM_ACM MSC; n = 15/18 for ACM CM_HC MSC; n = 15/18 for HC CM_ACM MSC. Co-cultures from three independent differentiations were used. Data information: mean + SEM. *P < 0.05, **P < 0.01 and ***P < *0.0001*.

The in silico study, which simulated CM-cMSC cell interactions, revealed potential cell-cell communication pathways and unique interactions (Fig. 4; Supplementary File Data S1-2). Cell-cell communication can occur through paracrine signalling, which involves soluble mediators. To address this issue from the perspective of the effective release of these mediators, we performed a secretome analysis of our co-cultures. Conditioned media were collected and analysed using Olink technology. Ninety-two factors involved in cardiovascular alterations were screened (Supplementary File Data S3), and 36 of these were found to be differentially secreted among the four co-cultures. The diversity of the secretomes from the four co-cultures is graphically shown in Fig. 5A, while the secretion levels of all 36 factors are shown in Supplementary Fig. S7. We focused on factors that could be involved in ACM-dependent phenotypic alterations. For instance, we identified factors related to cardiac remodelling and the balance between matrix synthesis and deposition, such as Collagen 1A1 (Col1a1), Matrix Metalloproteinases 9 (MMP9), and Metalloproteinase inhibitor 4 (TIMP4). The levels of secreted Col1a1, MMP9, and TIMP4 were increased in co-cultures containing ACM cMSC (Fig. 5B–D; Supplementary Fig. S7, Supplementary File Data S3). Additional factors differentially secreted and known to be involved in fibrosis and mechanosensory functions were urokinase-type plasminogen activator receptor (uPAR) and platelet endothelial cell adhesion molecule (PECAM-1) respectively. The levels of uPAR and PECAM-1 were elevated in the presence of ACM cMSC and HC cMSC respectively (Fig. 5E, F; Supplementary Fig. S7, Supplementary File Data S3).

Markers associated with muscle damage or ACM severity, such as myoglobin (MB), Growth Differentiation factor-15 (GDF-15), and factors involved in cardiac lipid metabolism, such as LDL receptor, were highly expressed in the conditioned media and differentially represented among the four co-cultures. GDF-15 and LDL receptor were increased in co-cultures containing ACM cMSC, whereas MB was more abundant in co-cultures with ACM CM (Fig. 5G–I; Supplementary Fig. S7, Supplementary File Data S3).

Notably, the DLK1 protein, previously identified in the in silico interactome studies, was found to be heavily regulated in the different conditioned media. Specifically, DLK1 was secreted exclusively by co-cultures containing HC CM (Fig. 5L; Supplementary Fig. S7, Supplementary File Data S3).

### DLK1 modulates fibro-adipose accumulation of ACM stromal cells

In silico interactome studies of CM-cMSC interactions have highlighted that the DLK1_NOTCH1 interaction was predicted only when the ligand was derived from CM and the interaction was reduced when CM were from ACM (Supplementary File Data S2-S3). Furthermore, data from secretome analysis indicated that DLK1 protein was present only in the supernatants of co-cultures including HC CM (Fig. 5L). Given the consistency between the two analyses, we decided to further investigate DLK1_NOTCH1 pathway.

**Fig. 6:**
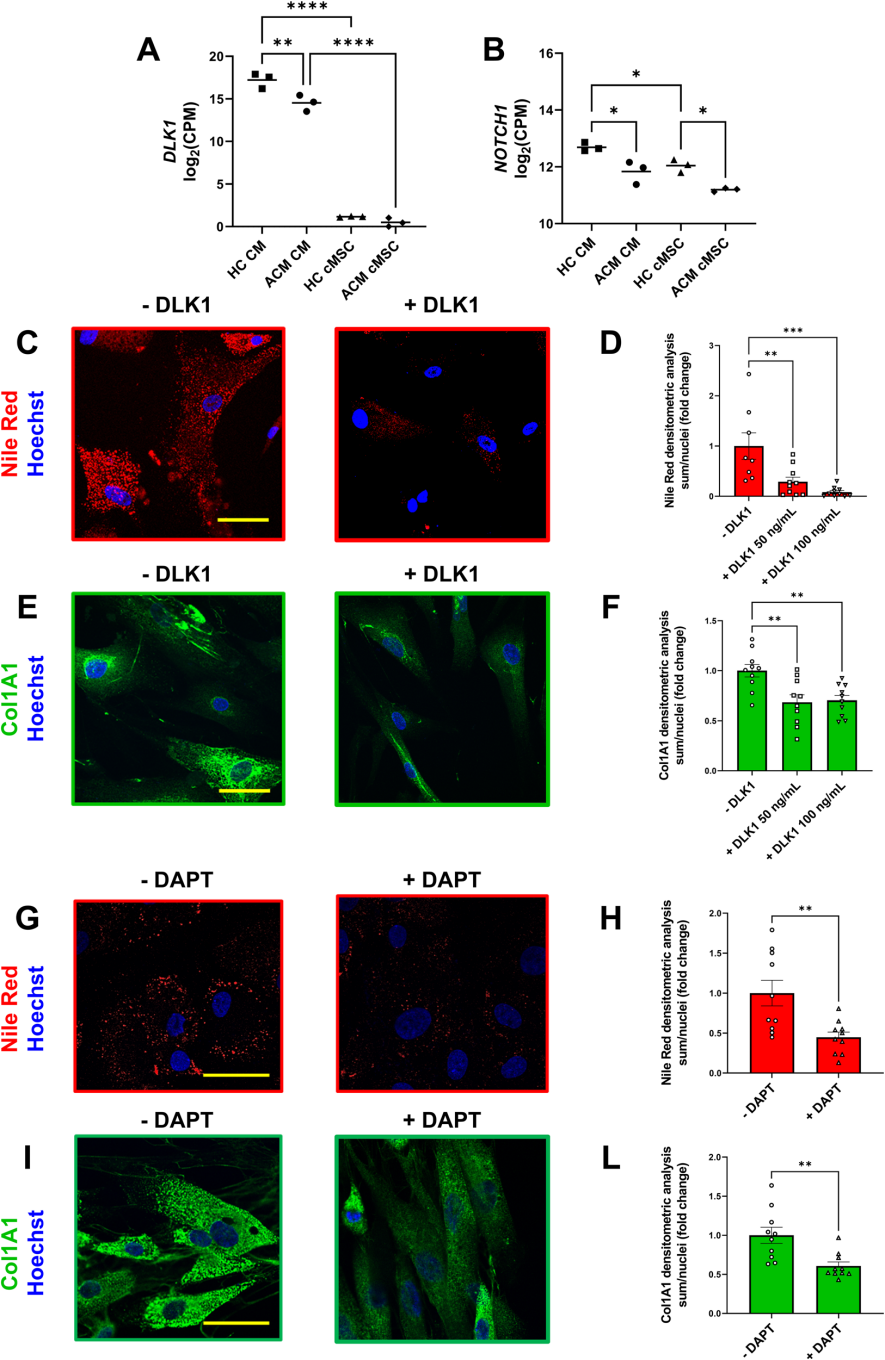
DLK1 expression and activity on ACM cMSC. **A**, **B** Expression levels of DLK1 and NOTCH1 in log2 counts per million reads (CPM) from transcriptome analysis of CM and cMSC from HC donors and ACM patients. *P*-values from the One-way ANOVA and Tukey’s post-test are indicated in each panel. Three batches of ACM cMSC were used. **C**, **G** Representative images of Nile Red staining of ACM cMSC cultured in adipogenic medium for three days, with or without supplementation (+/− DLK1 or /-DAPT) with DLK1 (50 ng/mL and 100 ng/mL) or DAPT (10 μmM). Nuclei are stained with Hoechst 33342. Magnification: 40X; scale bar:50 μM. **D**, **H** Quantification of Nile Red staining in ACM cMSC. The mean intensity of Nile Red is shown for DLK1 or DAPT treatment relative to the basal level of ACM cMSC in AM. *P*-values from One-way ANOVA and Tukey’s post-test are indicated. N = 8 for -DLK1; n = 10 for DLK1 50 ng/mL; n = 10 DLK1 100 ng/mL; n = 10 for -DAPT; n = 10 + DAPT. Three batches of ACM cMSC were used. **E**, **I** Representative images of Collagen immunostaining of ACM cMSC cultured in low serum growth medium (2% FBS) with TGF-β1 (5 ng/mL) for three days, with or without supplementation (+/− DLK1 or /-DAPT) with DLK1 (50 ng/mL and 100 ng/mL) or DAPT (10 μmM). Magnification:40X; scale bar:50 μM. **F**, **L** Quantification of Collagen immunostaining in ACM cMSC. The mean fluorescence intensity of Collagen is shown for DLK1 or DAPT treatment relative to the values of basal level of ACM cMSC in in GM low serum supplemented with TGF-β1 (BASE). *P*-values from One-way ANOVA and Tukey’s post-test are indicated in each panel. N = 10 for -DLK1; n = 10 for DLK1 50 ng/mL; n = 10 DLK1 100 ng/mL; n = 10 for -DAPT; n = 10 + DAPT. Three batches of ACM cMSC were used. Data information: mean + SEM. **P < 0.01 and ***P < 0.001.

Transcriptome data confirmed that DLK1 expression was restricted to CM and was higher in HC CM compared to ACM CM (Fig. 6A; Supplementary Fig. S4B). In contrast, *DLK1* expression levels were extremely low in stromal cells (Fig. 6A; Supplementary Fig. S4C). Notably, both CM and cMSC expressed the gene encoding for NOTCH1, a known receptor of DLK1 (Fig. 6B; Supplementary Fig. S4B, C). DLK1 is recognized as a negative regulator of adipogenesis [19] and has been implicated in the transition from fibroblasts to myofibroblasts [20]. We therefore hypothesized that the absence of DLK1 may contribute to the enhanced propensity of ACM cMSC to differentiate into adipocytes and myofibroblasts in vitro.

First, ACM cMSC were cultured in adipogenic medium in the presence or absence of recombinant DLK1. Following the treatment, Nile Red staining was performed to mark neutral lipids (Fig. 6C, D). The presence of DLK1 reduced lipid accumulation in ACM cMSC compared to adipogenic medium only (Fig. 6C, D). Next, we investigated DKL1’s involvement in ACM cMSC-mediated collagen production by culturing ACM cMSC in a pro-fibrotic medium and treated with DLK1 (Fig. 6E, F). Immunofluorescence analysis demonstrated that DLK1 treatment reduced the production of Collagen I in ACM cMSC (Fig. 6E, F). These results underscore the crucial inhibitory role of DLK1 typically secreted by HC CM, in regulating the adipose and pro-fibrotic differentiation of ACM cMSC.

To further validate that DLK1 modulates fibro-adipose accumulation through inhibition of the NOTCH pathway, we treated ACM cMSC with the gamma secretase inhibitor DAPT, known as an inhibitor of the NOTCH signalling pathway [21]. DAPT treatment, similarly to DLK1 treatment, was able to reduce fibro-adipose accumulation in ACM cMSC (Fig. 6G–L).

We believe this effect is particularly relevant in ACM cMSC, where there is a genetic predisposition towards fibro-adipose differentiation. These results could explain the greater differentiation capacity observed in co-cultures containing both ACM CM and ACM cMSC, compared to those where CM were HC.

## Discussion

Recent advances in understanding the mechanisms of ACM suggest that it is a multifaceted condition involving different cell types. Our study significantly contributes to the knowledge of ACM pathogenesis by demonstrating that cell-specific phenotypes are not solely attributable to the intrinsic properties of each cell type. Instead, these phenotypes are fundamentally influenced by interactions with surrounding cardiac cells.

To fully understand the crosstalk between CM and cMSC, we used different combinations of mutated or healthy cells (Fig.1). The genetically homogeneous co-cultures replicate naturally occurring conditions (all HC and ACM), while the other two are artificial combinations that would never occur in a patient but are instrumental in understanding the relative contribution of mutated cells to overall derangements. Our multicellular model was intentionally kept as simple as possible, facilitating both direct and paracrine interactions, and ensuring easy-to-read and reproducible 2D readouts. The limitation posed by the immaturity of hiPSC CM in this model is mitigated by the presence of cMSC and longer culturing [22]. Other in vitro ACM models have been previously published but they only partially recapitulate the disease phenotypes [22, 23].

There are limited insights in the literature on the role of extracellular factors in the pathogenicity of cell behaviour, particularly in how they facilitate disease progression in cMSC. Various molecules- such as TGFβ1, oxLDL, NPY, inflammatory cytokines, cyclophilin A- have been shown to exacerbate the cMSC phenotype [9, 24–27]. We demonstrated that co-culture with ACM CM can trigger fibro-adipose differentiation in stromal cells (Fig. 2). Importantly, ACM cMSC, which are sometimes overlooked in the context of ACM pathogenesis [28], can contribute to CM dysfunctions (Fig. 3). Therefore, cMSC play both an active direct role on cardiac substrate remodelling and an active indirect role on CM-dependent functions.

Our models were capable of detecting rhythm dysfunctions through video recordings of culture movement, reflecting both cardiomyocyte-dependent arrhythmias and the influence of surrounding cMSC. However, we acknowledge that we did not use a direct method of arrhythmia analysis (Fig. 3). In ACM, two primary mechanisms of arrhythmia are described: “focal” (cell-based) and “re-entry” (tissue-based) arrhythmogenesis. The former, prevalent during the early, ‘concealed’ phase of ACM, primarily involves intrinsic calcium-handling defects in cardiomyocytes, leading to early or delayed after-depolarization (EADs and DADs) [29–32]. The latter mechanism becomes evident in advanced stages of ACM, where extensive structural alterations (fibro-fatty accumulation, mainly caused by cMSC) slow down electrical signal conduction, contributing to re-entrant circuits [33]. Accordingly, our co-cultures exhibited a higher number of aftercontractions (the contractile counterpart of afterpotentials) in the ACM CM_ACM cMSC group compared to the ACM CM_HC cMSC group (Fig. 3C). Additionally, incomplete relaxation was observed in our monolayers when HC stromal cells were replaced with ACM stromal cells, possibly due to calcium accumulation (Fig. 3C). This suggests that ACM cMSC dysregulation may play a fundamental role both in focal triggered activity and, over the long term, as a source of tissue discontinuity.

Cell-cell influence occurs either through direct or paracrine interaction between ligands and receptors [34]. In silico interactome theoretically identifies both direct and paracrine signals. Our results indicated that the most differentially identified communications were paracrine (ligand-receptor) rather than direct (between membrane proteins; Supplemental data file S1-2). Regarding direct interactions, it has been reported that CX43 expression and localization are altered in ACM [22, 35, 36]. CX43 mediates both electric coupling, potentially leading to crucial excitation and conduction disturbances of pro-arrhythmic significance [37], and the direct passage of small molecules. However, our analyses did not identify mutation-induced differential interactions of hemichannels between cells pairs (Supplemental data file S1-2). Other known mechanisms of direct cell-cell interaction in ACM [16] may arise from mechanical stimuli, resulting in mechanotransduction signals that eventually regulate cell conduct. We cannot exclude the possibility that in our co-cultures, differences in substrate stiffness or altered contractile patterns caused by the ACM genotype in one cell type do not influence the other cell type.

Among the detected paracrine interactions, significant interest was raised by the DLK1_NOTCH1-2-3-4 pathway, which was more pronounced in conditions where CM were healthy and inhibited fibro-adipose remodelling (Fig. 4, Supplementary Figs. S5, 6, Supplemental data file S1-2). This protein is known to regulate adipogenesis, specifically the transition of cMSC to adipocytes [19]. DLK1 acts through different pathways, during stromal cell clonal expansion, depending on whether it is secreted or retained on the cell membrane, leading to a differentiation block of stromal cells into adipocytes [19]. The role of DLK1 in fibrosis is controversial, but in cardiac cells, it has been reported to inhibit the process [20], which aligns with our data.

As shown in Fig. 4B and Supplementary Fig. S6 and evident from Supplemental data file S2, ACM CM lose communication with HC cMSC mediated by Collagen 3 and 5 and different integrin receptors, which are abundant in HC CM. However, a large set of signals involving different minor forms of collagens and a1b1 or a11b1 integrin receptors were established. This reframing suggests a different role for these proteins as signalling molecules versus their function in extracellular matrix reorganization. ACM CM developed new interactions with HC cMSC, such as TGFB2_TGFBR1 and IL6_IL6R. Both the TGFβ pathway [9, 38] and the inflammatory cytokines response [39, 40] are known mediators of pro-fibrotic messages. Accordingly, ACM CM send novel signals to cMSC, such as FGF1 and FGF18, which typically mediate fibroblast and pre-adipocyte proliferation [41, 42]. Notably, ephrin-ephrin receptor interactions were lost in ACM CMs. Dysregulation of this signalling pathway has already been described in ACM cells [43].

Regarding the signals sent from cMSC (Fig. 4A, Supplementary Fig. S5, Supplemental data file S1), ACM-specific communications included IL6 and its receptor, highlighting the importance of inflammatory cytokines, and TGFB3_TGFBR3, which may promote fibrosis. In addition, WNT5A ligand, interacting with EPHA7 or ROR2, was exclusively present in ACM cMSC, while the interaction of WNT1 and WNT4 with FZD8, observed in HC cMSC, was abolished. Indeed, both canonical and non-canonical WNT signalling pathways have been extensively characterized as mediators of ACM [17, 44–46]. Furthermore, ACM cMSC exhibited a loss of communication between PTN (Pleiotrophin) and different receptors such as PLXNB2, PTPRS, PTPRZ1 and ALK, which are likely involved in regulating CM proliferation and survival [47–49].

A targeted proteomic approach allowed us to validate some of the predicted communications by identifying factors that were effectively secreted by the different co-cultures (Fig. 5A and Supplementary Fig. S7).

COL1A1, a stiff collagen [50], was mainly secreted by the ACM stromal cells in the co-cultures, with the highest levels observed in the ACM cMSC combinations (Fig. 5B). This aligns with our immunostaining results shown in Fig. 2A and previously published data [9, 51]. MMP-9, an enzyme involved in the remodelling of the extracellular matrix through the degradation of collagen, elastin, fibronectin, gelatin, and laminin [52], was abundant in co-cultures where stromal cells were of the ACM type (Fig. 5C). TIMP4, a metalloproteinase inhibitor that regulates extracellular matrix balance [53], was also abundant in ACM co-cultures, likely secreted primarily by cMSC (Fig. 5D). TIMP4 was found to be poorly expressed in ACM heart tissue, as well as in other failing hearts, but is abundant in control hearts [54]. Conversely, its circulating expression is higher than in controls in patients with pro-fibrotic conditions such as idiopathic pulmonary fibrosis [55], though it has never been tested in ACM. uPAR, part of a proteolytic system relevant for fibrinolysis and extracellular matrix homeostasis, plays a key role in the progression or degradation of fibrosis and was also found to be abundant in ACM co-cultures (Fig. 5E) [56]. Platelet endothelial cell adhesion molecule (PECAM-1), a regulator of mechanosensory functions, is associated with left ventricular dilation and systolic dysfunction in mice when absent [57] (Fig. 5F).

GDF-15 [58], member of the TGF-β superfamily, is prominently induced by “injury” [59] and has been previously reported to predict biventricular involvement in ACM [60]. In line with these findings, it was found to be abundant in co-cultures containing ACM cMSC (Fig. 5G).

Among the proteins enriched in the co-cultures containing ACM CM, MB is particularly noteworthy (Fig. 5H). MB is one of the most abundant proteins in muscle cells and is released upon injury, serving as a marker of myocardial damage in cardiomyopathies and myocarditis [61–63]. Its increase suggests a higher rate of cell death in co-cultures including ACM CMs.

The receptor for LDL was highly expressed in the conditioned media when ACM cMSC were present (Fig. 5I). While its role in ACM has never been studied, lipid metabolism derangements in ACM [26] suggests a possible involvement.

The identification of factors deemed relevant in the in silico interactome converged in the secretome analysis, as seen, for instance, with DLK1 (Fig. 5L). The decision to validate this pathway is due to its recognised role in the regulation of adipogenesis and fibrosis, known hallmarks of ACM, and the novelty of its possible involvement in the disease pathogenesis. Both ectopic DLK1 administration and NOTCH inhibition by DAPT led to a reduction in fibro-adipose accumulation (Fig. 6), suggesting that the modulation of the newly discovered DLK1-NOTCH pathway may regulate fibro-adipose differentiation of stromal cells in ACM. Future gene-editing experiments could confirm these results and the action of DLK1 through NOTCH.

Given the progressive propensity toward fibro-adipose differentiation of co-cultures as HC cMSC and/or HC CM are replaced by ACM counterparts (Fig. 2), we speculate that the mechanisms determined by the mutation in *PKP2* and those due to the absence of DLK1 are independent and additive phenomena.

It is known that inflammatory components contribute to ACM pathogenesis. We acknowledge that our simplified model cannot account for the contribution of inflammatory cells. However, we were able to observe the effect of pro-inflammatory mediators secreted either by CM or cMSC.

In conclusion, we developed a new, simple, multicellular ACM model to simulate the early/intermediate stages of phenotype development. This model has proven valuable in understanding both cell-autonomous and crosstalk-mediated cell behaviours, as well as the underlying pathways in ACM.

Deciphering the factors contributing to disease progression is crucial for devising effective therapeutic strategies. Indeed, DLK1 can be considered as a potential therapeutic target to moderate ACM pathogenesis. This hypothesis will require further preclinical studies on complex cultures and mice. Furthermore, this approach can be extended to investigate other genetic cardiomyopathies or other cell type pairings, aiding in the unravelling of genotype-specific cell characteristics and cell-cell interactions.

## Materials and methods

### Ethical statement

This study complies with the declaration of Helsinki and was approved by the Istituto Europeo di Oncologia and Centro Cardiologico Monzino IRCCSs Ethics Committee (R1020/19-CCM1072; date of approval: 3/7/2019). Written and informed consent was obtained from all participants. Supplementary Tables S1, S2 summarize the features of cMSC and hiPSC cell lines, respectively.

### Patient cells

A right ventricle (RV) biopsy was obtained during a diagnostic procedure [64] from a patient affected by ACM and carrying the heterozygous c.2013delC *PKP2* mutation as previously described [8].

Fibroblasts were obtained from a skin biopsy of the same ACM patient and reprogrammed into hiPSC. *The PKP2* mutation was corrected using CRISPR/Cas9 technology to create an isogenic hiPSC line, as described in [65].

### Isolation and culture of primary cardiac stromal cells

cMSC were isolated from RV biopsy and cultured as previously described [8, 66]. Briefly, ventricular biopsies were washed with PBS, cut into 2–3 mm pieces, and incubated at 37 °C for 1.5 h under continuous agitation in Iscove’s Modified Dulbecco’s media (IMDM; Gibco/Thermo-Fisher, Waltham, MA, USA) containing 3 mg/mL collagenase NB4 (Serva, Heidelberg, Germany). The digested solution was then centrifuged at 400 g for 10 min, washed with PBS, and centrifuged again. The obtained pellet was resuspended in growth medium (GM) consisting of IMDM supplemented with 20% fetal bovine serum (FBS; Euroclone, Pero (MI), Italy), 10 ng/mL basic fibroblast growth factor (R&D Systems, Minneapolis, MN, USA), 10,000 U/mL penicillin (Invitrogen/Thermo-Fisher, Waltham, MA, USA), 10,000 µg/mL streptomycin (Invitrogen/Thermo-Fisher, Waltham, MA, USA), and 20 mmol/L L-Glutamine (Sigma-Aldrich, Saint Louis, MO, USA). The cells were seeded onto uncoated Petri dishes (Corning, New York, NY, USA) and non-adherent cells were removed after 24 h. cMSC were used for the experiments between passage 3 and 8, and mycoplasma contamination was excluded.

### Differentiation of hiPSC into cardiomyocytes

hiPSC-CM cells were generated from undifferentiated hiPSC through the induction cardiac mesoderm as previously described [67]. Mycoplasma-free hiPSC were used to induce CM differentiation. Briefly, 25,000 cells per cm^2^ were seeded on Matrigel on day −1. On day 0, cardiac mesoderm was induced by BPEL medium [68] supplemented with a mixture of cytokines [20 ng/mL BMP4, (R&D Systems, Minneapolis, MN, USA); 20 ng/mL ACTIVIN A, (Miltenyi Biotec, Bergisch Gladbach, Germany); 1.5 μM GSK3 inhibitor CHIR99021 (Selleckchem, Houston, TX, USA)]. After 3 days, the cytokines were removed and the WNT inhibitor XAV939 (5 μM, Tocris, Bristol, UK) was added for 3 days. On day 6, medium was replaced with BPEL medium without supplement. The medium was refreshed every 2-3 days until the beating cells appeared. Metabolic selection of hiPSC-CM with 4 mM sodium-L-lactate (Sigma-Aldrich, Saint Louis, MO, USA) was performed twice, for 2 days each, to maximize CM enrichment [67]. After selection, the lactate medium was replaced with BPEL.

### Co-culture assembly

On the day of co-culture formation (day 0), cMSC were detached using Trypsin 1X for 5 min at 37 °C, 5% CO_2_, centrifuged for 5 min at 400 × *g*, resuspended in BPEL medium and counted. hiPSC-CM at day 14–21 (HC CM, ACM CM) were dissociated using the Multi Tissue Dissociation Kit 3 (Miltenyi Biotec, Bergisch Gladbach, Germany) according to the manufacturer’s instructions, resuspended in BPEL medium and counted. Cardiac co-cultures (Fig. 1A) were assembled by combining 85% hiPSC-CM and 15% primary cMSC, as previously tested [22] to a total of 150,000 cells per 1 mL BPEL in four different combinations (Fig. 1B). To differentiate the four co-cultures graphically, a colour code was assigned to each: white for the co-culture composed of healthy control-cardiomyocytes and healthy control-stromal cells (HC CM_HC cMSC); blue for the co-culture composed of healthy control-cardiomyocytes and arrhythmogenic cardiomyopathy-stromal cells (HC CM_ACM cMSC), green for the co-culture composed by arrhythmogenic cardiomyopathy-cardiomyocytes and healthy control-stromal cells (ACM CM_HC cMSC); and red for the co-culture composed by arrhythmogenic cardiomyopathy-cardiomyocytes and arrhythmogenic cardiomyopathy-stromal cells (ACM cMSC_ACM CM).

For all co-cultures, cell suspensions were seeded on Matrigel-coated coverslips placed in 24-well microplates and incubated at 37 °C, 5% CO_2_ for 10 days with half of the media refreshed on day 5. Functional analysis of the co-cultures was performed on day 10.

### Stromal cell differentiation and treatment

To induce adipogenic differentiation, cells were plated at a density of 50,000 cells/cm^2^ and maintained in adipogenic medium, consisting of IMDM supplemented with 10% FBS (Euroclone, Milan, Italy), 0.5 mmol/L of 3-isobutyl-1-methylxanthine (Sigma-Aldrich, Saint Louis, MO, USA), 1 µmol/L of hydrocortisone (Sigma-Aldrich, Saint Louis, MO, USA), 0.1 mmol/L of indomethacin (Sigma-Aldrich, Saint Louis, MO, USA), 10,000 U/mL of penicillin (Invitrogen/Thermo-Fisher, Waltham, MA, USA), 10,000 µg/mL of streptomycin (Invitrogen/Thermo-Fisher, Waltham, MA, USA), and 20 mmol/L of L-Glutamine (Sigma-Aldrich, Saint Louis, MO, USA) [51].

To induce the pro-fibrotic differentiation, cells were plated at a density of 50,000 cells/cm^2^ and treated with 5 ng/mL of TGF-β1 (PeproTech, Cranbury, NJ, USA) following overnight growth in low-serum growth medium (2% FBS) [51]. Adipogenic and pro-fibrotic differentiation were assessed after 3 days. For experiments involving DLK1 and DAPT (Abcam, Cambridge, UK) the cells were treated with serial dilutions (0–100 ng/mL range) of recombinant DLK1 [69] or 10 μmM of DAPT [21]. DLK1 or DAPT was added daily for 3 days.

### Detection of cell fibro-adipose accumulation

To detect fibro-adipose accumulation, co-cultured monolayers or single cell population were fixed using 4% paraformaldehyde (Santa Cruz biotechnology, Dallas, TX, USA) for 10 min. This was followed by incubation with PBS supplemented with 5% BSA and 0.1% Triton X-100 (PBS-T/BSA) for 60 min to block nonspecific binding sites. All co-culture monolayers were incubated overnight at 4 °C with specific primary antibody against α-actinin to visualize CM population. To detect fibrotic accumulation, co-culture monolayers were co-stained with a collagen antibody (as reported in Supplementary Table S3). Subsequently, the slides were incubated for 1 h at room temperature (RT) with specific fluorescence-labelled secondary antibodies (Invitrogen/Thermo-Fisher, Waltham, MA, USA). For the detection of lipid accumulation, co-cultured monolayers were co-stained with 12.5 ng/mL of Nile Red (Invitrogen/Thermo-Fisher, Waltham, MA, USA) for 1 h at RT during the incubation with secondary antibody. Nuclei were stained using Hoechst 33342 (Sigma-Aldrich, Saint Louis, MO, USA). To detect fibro-adipose accumulation in cMSC following specific differentiation induction and treatments (described in the preceding section), slides were incubated with either collagen or Nile Red as described above. Images were acquired using a confocal microscope in Z-stack mode with a 40× oil immersion objective (LSM710-ConfoCor3 LSM, Zeiss, Oberkochen, Germany) and the Zen 2008 software (Zeiss, Oberkochen, Germany). Optical sections were captured at a resolution of 1,024 × 1,024 pixels, with the pinhole diameter adjusted to 1 Airy unit for each emission channel. Quantitative image analysis was performed by scanning 5-10 randomly selected fields, each containing 15-30 cells each, while maintaining consistent acquisition parameters (laser power and detector amplification). These parameters were below the pixel saturation between the samples of interest and the negative controls. Fluorescence signal quantification was performed using ImageJ software (National Institutes of Health, Bethesda, MD, USA) on Z-Stack images. Each image was separated into individual channels, and converted into 8-bit grayscale images to subtract background noise. The fluorescence intensity (relative to each channel) and the nuclei count for each field were measured using ImageJ tools. The fluorescence intensity of each channel in each field was normalized to the corresponding number of nuclei in the field.

### Contractility measurements

Movies of beating monolayers of co-cultured hiPSC-CM and cMSC were recorded at 100 frames per second with a Kiralux CS135MUN camera (Thorlabs, Newton, NJ, USA) at full resolution. The camera was mounted on an ECLIPSE TE-200 microscope (Nikon, Tokyo, Japan) and a 40X air objective was used. The measurements were performed at physiological temperature (~37 °C), with temperature control provided by an in-line solution heater (SH-27B, Warner Instruments, Holliston, MA, USA) positioned in close proximity to the monolayers. The monolayers were stimulated through a pair of gold electrodes positioned near -but not touching- the cell monolayer. The pulses were triggered by an external stimulator (3165 Multiplexing Pulse Booster – Ugo Basile, Gemonio (VA), Italy) connected to a Molecular Devices Digidata 1440A controlled by pClamp 10 (Molecular Devices, San Jose, CA, USA). Different pacing frequencies were used: Spontaneous, 0.5 Hz, 1 Hz, 2 Hz.

The extracellular modified Tyrode’s solution used for contraction analysis contained (in mM): D-(+)-glucose 10, HEPES 5.0, NaCl 140, KCl 5.4, MgCl_2_ 1.2, CaCl_2_ 1.8, pH adjusted to 7.3 with NaOH (Tyrode’s solution reagents were purchased from Sigma-Aldrich, Saint Louis, MO, USA).

### Contractility analysis

Contractility was analysed from a total of n = 322 raw AVI movies, distributed over three independent batches of differentiation, using MUSCLEMOTION [18, 70]. The movies were combined through a custom-made R script (https://github.com/l-sala/Thorlabs_Kiralux_Concatenate_Files, Thorlabs, Newton, NJ, USA). The protocol for acquiring movies for contraction analyses was as follows: 20 s of electrical stimulation prior to recording to allow the monolayers to reach a steady-stable contractions, followed by 20 s of recording.

Initially, we qualitatively evaluated the contraction patterns in cell monolayers, observing several abnormalities. Although it is impossible to relate these abnormalities directly to arrhythmias emerging in clinical settings, these phenomena are likely of arrhythmic significance. Such events include the following:

- Failure to follow the imposed pacing frequency, due to overriding spontaneous firing.
- Incomplete relaxation or increased diastolic tension, likely indicating intracellular Ca^2+^ accumulation.
- Presence of aftercontractions, which are indicators of spontaneous Ca^2+^ release events and proxies of arrhythmogenic afterpotentials.
- Presence of alternating contraction amplitude, which is commonly associated with arrhythmogenic electrical alternans or decreasing peak amplitude.
- Presence of chaotic contraction patterns, suggestive of fibrillation.
- Different combinations of the above.

Representative examples from each subtype are presented in Supplementary Fig. S1.

Next, in the monolayers that displayed a “normal” contractile pattern, we performed a quantitative analysis of their temporal parameters. Specifically, Contraction Duration and Normalized Contraction Amplitude were evaluated at several pacing rates.

### RNA-Seq data and analysis of cell−cell interactions

Homogeneous cell cultures (ACM CM, HC CM, ACM cMSC, HC CM, separately, 3 batches of differentiation for CM and 3 batches of cMSC, each type) were lysed in RL lysis buffer (Norgen Biotek corp., Thorold, Canada). RNA was isolated from cells using a Total RNA Purification kit (Norgen Biotek corp., Thorold, Canada). The quantification of the isolated RNA was determined by Qubit™ 4 Fluorometer (Invitrogen/Thermo-Fisher, Waltham, MA, USA) and a quality check was performed using the Bioanalyzer (Agilent, Santa Clara, CA, USA). The RNA samples that passed the quality check were then sent to Eurofins Ltd Genomic Service for sequencing (Eurofins, Milan, Italy).

RNA libraries were prepared for transcriptome sequencing using standard Illumina protocols as part of the INVIEW Transcriptome Discover protocol. For library preparation, mRNA was fragmented, and random hexamer-primed cDNA synthesis was performed. Paired-end read sequencing (2 ×150 bp) was performed on the Illumina HiSeq 4000. Reads were aligned to the human genome reference (GRCh38/hg38) using BWA software (v0.7.1), and mapped reads were used to quantify gene expression with FeatureCounts (v2.0.0).

The resulting gene expression matrix and the vector containing sample information were imported into the R environment (v4.1.1). Using the DaMiRseq package [71], genes with read counts lower than 5 for at least 50% of samples were filtered out, and the data converted to log2 counts per million mapped reads (CPM). Differential analyses were performed using the ‘limma’ R package [72].

Cell-cell communication analysis was conducted using the CellPhoneDB package (version 3.1.0) [73]. The investigation focused on discerning enriched interactions between two distinct cell types (CM and cMSC) either healthy or ACM. CellPhoneDB outputs included interaction scores based on the expression levels of ligands and receptors, as well as *p*-values indicating the likelihood of cell-type specificity of a given receptor-ligand complex. These *p*-values were calculated after random permutation, based on the proportion of means that are greater than or equal to the actual mean. We reported in Fig. 4, Supplementary Data file S1, S2 and Supplementary Figs. S5, S6 only the statistically significant interactions. The selection of biologically relevant receptor-ligand or receptor-receptor pairs was carried out through a manual curation process based on the significance of pairs and their possible relevance in ACM pathogenesis.

### Proteomic profiling of soluble factors in conditioned media

Conditioned media were generated by harvesting medium from co-cultures on day 10 to collect all soluble factors released during hiPSC-CM-cMSC interactions. The conditioned media were quantified using the Olink multiplex proximity technology Cardiovascular III panel, which includes 92 targets (Bioscience AB, Uppsala Sweden). Briefly, each protein was detected by oligonucleotide-labelled antibodies binding to their complementary target proteins. When two antibodies are in close proximity, a new protein-specific DNA reporter sequence is formed through a proximity-dependent DNA polymerization reaction. The resulting sequence is then amplified and quantified by standard real-time PCR. The relative amounts of protein were quantified as normalized protein expression (NPX), where an increase of 1 NPX represents a doubling of the relative protein concentration. Further information on the Olink assay is available at http://www.olink.com (Olink Bioscience AB, Uppsala Sweden). Figure 5A show the results of the analysis in terms of the two most informative projections from a multiple-dimensional scaling analysis of the samples and represents the diversity of the secretomes from the four co-cultures. Specifically, Multi-Dimensional Scaling (MDS) allow to represent the dissimilarities between the sample proteomes in a two-dimensional space. Therefore, to avoid introducing noise by data imputation, only the samples with complete proteomes were plotted and to exclude the samples with more than 20% of missing values randomly distributed. Instead, for the statistical analysis on a single protein (Fig. 5B–L), all samples with an expression value were used.

### Statistical analysis

Continuous variables are presented as mean ± standard error (SEM), and categorical data as counts and proportions. A normality test was performed for each sample variable. Normally distributed continuous variables were compared using Student’s *t* test for independent samples. Comparisons among three or more groups were conducted with one-way ANOVA or Kruskal-Wallis test, as appropriate, in conjunction with Tukey’s multiple comparison post-hoc tests. The proportions of the frequencies of categorical variables was compared using Fisher’s exact test, given the sample size, with the ‘fisher.test()‘ function. Pairwise comparisons were made between the HC CM_HC cMSC group and the other co-culture subtypes. *P*-values were adjusted for multiple comparisons using the ‘p.adjust()‘ function with the Holm method [74]. A *p*-value < 0.05 was considered statistically significant, unless otherwise indicated. The statistical evaluation of outliers was performed before exclusion. Statistical analyses and graphics were done with either R (version 4.3.2) or GraphPad Prism 9.

## Supplementary information

Video S1

Video S2

Video S3

Video S4

Supplementary materials

## Data Availability

The data supporting the findings of this study are available within the article and its Supplementary Materials. Additional supporting data are available from the corresponding author upon reasonable request. The transcriptome data are available in the GEO repository (ID: GSE249301).
