# Supplementary material for: Cardiomyocyte and stromal cell cross-talk influences the pathogenesis of arrhythmogenic cardiomyopathy: a multi-level analysis uncovers DLK1-NOTCH pathway role in fibro-adipose remodelling
Source: Cell Death Discov. 2024 Nov 28;10:484. doi: 10.1038/s41420-024-02232-8 (PMC11604953; doi:10.1038/s41420-024-02232-8)
Supplement: Supplementary file 5 — Supplementary materials [file 41420_2024_2232_MOESM5_ESM.pdf]

Title

Cardiomyocyte and stromal cell cross-talk influences the pathogenesis of arrhythmogenic cardiomyopathy: a multi-level analysis uncovers DLK1-NOTCH pathway role in fibro-adipose remodelling

Running title: Cardiomyocyte and stromal cell cross-talk in ACM

Authors

Angela Serena Maione[1, *], Lara Iengo[1], Luca Sala[2,3], Ilaria Massaiu[4], Mattia Chiesa[5,6], Melania Lippi[1], Stefania Ghilardi[7], Chiara Florindi[3], Francesco Lodola[3], Antonio Zaza[3], Claudio Tondo[8,9], Marco Schiavone[8,10], Cristina Banfi[7], Giulio Pompilio[1,9], Paolo Poggio[4,9], Elena Sommariva[1].

Affiliations

[1] Unit of Vascular Biology and Regenerative Medicine, Centro Cardiologico Monzino IRCCS, 20138, Milan, Italy.
[2] Istituto Auxologico Italiano IRCCS, Center for Cardiac Arrhythmias of Genetic Origin and Laboratory of Cardiovascular Genetics, 20095, Milan, Italy.
[3] Department of Biotechnology and Biosciences, University of Milano-Bicocca, Milan, 20126, Italy.
[4] Unit for the Study of Aortic, Valvular and Coronary Pathologies, Centro Cardiologico Monzino IRCCS, 20138 Milan, Italy.
[5] Bioinformatics and Artificial Intelligence Facility, Centro Cardiologico Monzino IRCCS, 20138, Milan, Italy.
[6] Department of Electronics, Information and Biomedical Engineering, Politecnico di Milano, 20133, Milan, Italy.
[7] Unit of Functional Proteomics, Metabolomics, and Network Analysis, Centro Cardiologico Monzino IRCCS, 20138 Milan, Italy.
[8] Department of Clinical Electrophysiology and Cardiac Pacing, Centro Cardiologico Monzino IRCCS, 20138, Milan, Italy.
[9] Department of Biomedical, Surgical and Dental Sciences, Università degli Studi di Milano, 20122, Milan, Italy.
[10] Department of Systems Medicine, University of Rome Tor Vergata, 00133, Rome, Italy.

* Corresponding author. Angela Serena Maione, Centro Cardiologico Monzino IRCCS, Via Parea 4, 20138, Milan, Italy. Tel.: +39 02 5800 2771, Email: angela.maione@cardiologicomonzino.it

Supplementary figures

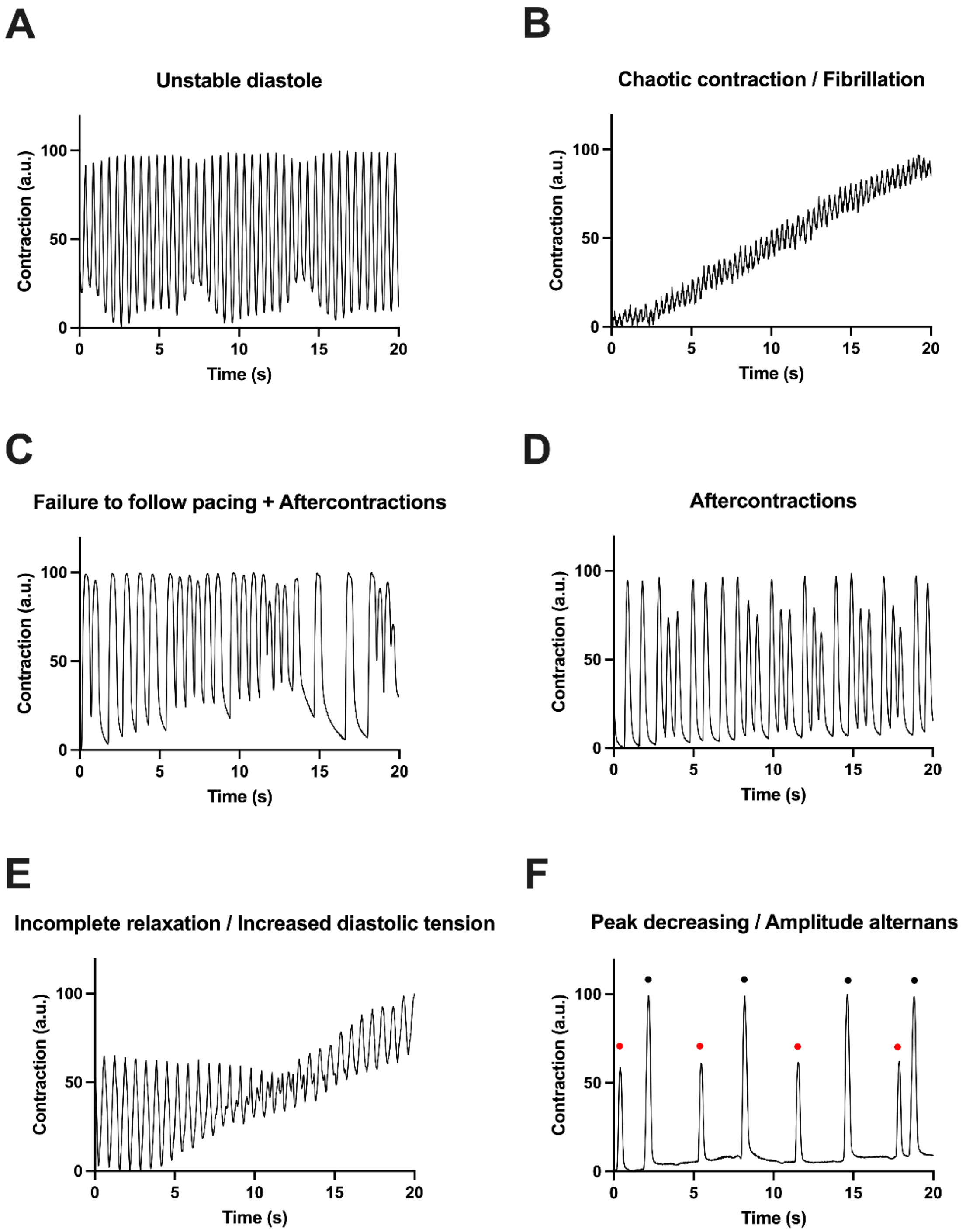

Figure S1: Examples of traces considered arrhythmic in the contraction analysis.

Normalized contraction profiles exhibiting **(A)** unstable diastole, **(B)** chaotic contraction / fibrillation, **(C)** failure to follow pacing and aftercontractions, **(D)** aftercontractions, **(E)** Incomplete relaxation or increased diastolic tension, **(F)** Peak decreasing and amplitude alternans; red and black dots indicate contraction amplitude alternans.

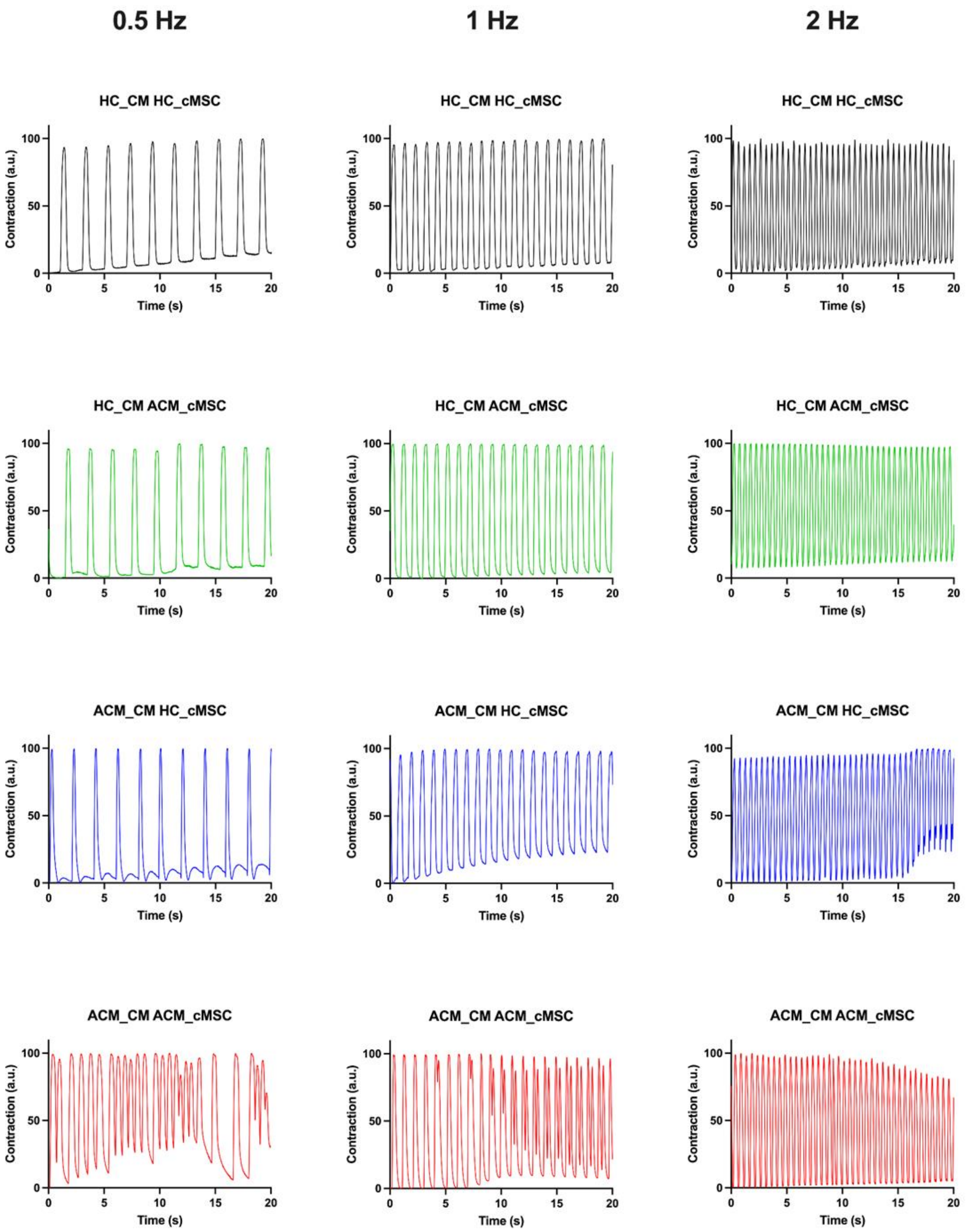

Figure S2. Representative traces at each pacing frequency.

Representative contraction profiles obtained with MUSCLEMOTION from the same cocultured monolayer paced at 0.5 Hz (left), 1 Hz (center) and 2 Hz (right). Examples include contractile events of likely arrhythmic significance like aftercontractions, decreasing peaks, Incomplete relaxation / increased diastolic tension.

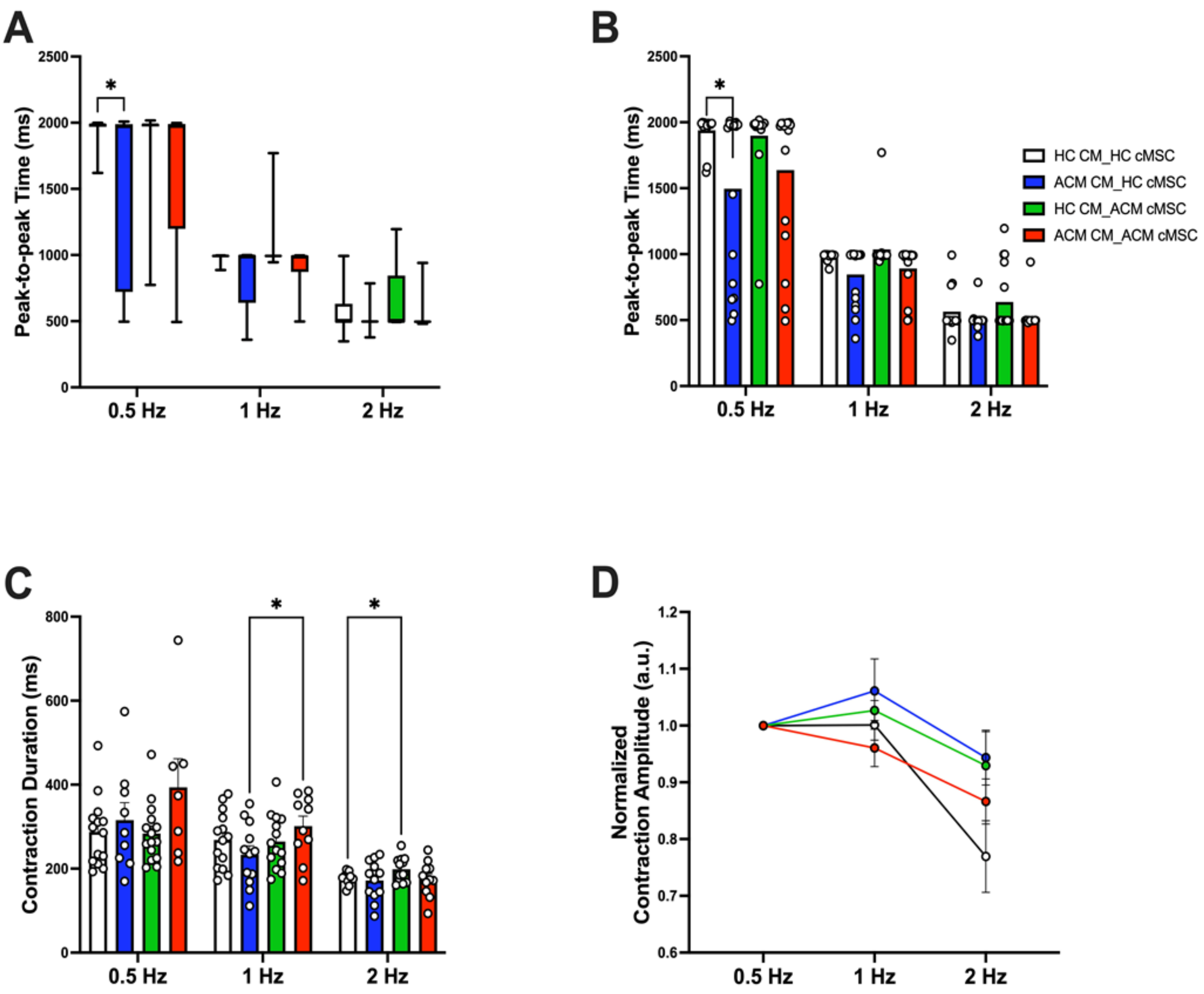

Figure S3. Temporal parameters measured from cocultured monolayers.

(A) Box-and-whiskers plot of the time between consecutive peaks obtained in the contractility plot (peak-to-peak time) at multiple pacing frequencies. **(B)** Bar graph with individual data points of the time between consecutive peaks obtained in the contractility plot (peak-to-peak time) at multiple pacing frequencies. Data points are clustered around the values corresponding to the imposed pacing frequency. Data points that deviate from the imposed pacing frequency are considered of arrhythmic significance. **(C)** Contraction duration at multiple pacing frequencies on cocultured monolayers exhibiting “Normal” contractile behaviour. **(D)** Normalized contraction amplitude values at multiple pacing frequencies on cocultured monolayers exhibiting “Normal” contractile behaviour. Due to the presence of missing values in the dataset as a consequence of monolayers starting to exhibit abnormal contractile behaviours, particularly at high pacing frequencies, the statistical significance was assessed with a mixed-effects model followed by a Dunnet’s multiple comparison post-hoc test among groups within each frequency. The asterisk (*) indicates $p < 0.05$. N = 3-17 per group. Temporal parameters were automatically obtained with MUSCLEMOTION.

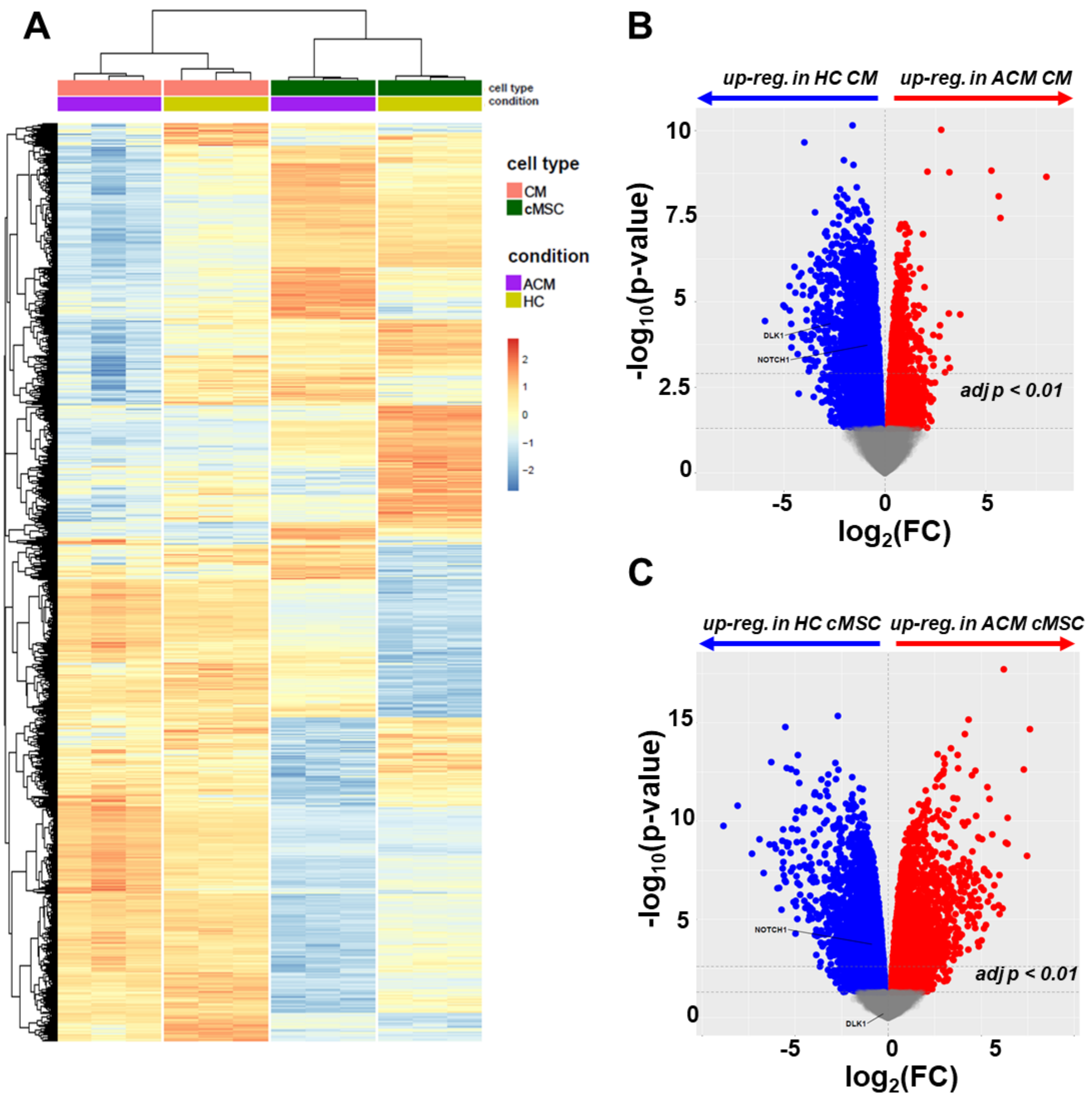

Figure S4. Differential gene expression analysis

(A) Heatmap showing that the unsupervised clustering, based on the most differentially expressed genes (ANOVA p-value < 0.01), allowed complete separation between 4 groups (ACM CM, HC CM, ACM cMSC, HC cMSC). Gene expression level was expressed as normalized value after row standardization and displayed as gradient colors from higher (dark red) to lower (dark blue). **(B)** Volcano plot of differential gene expression analysis, comparing ACM vs. HC CM. **(C)** Volcano plot of differential gene expression analysis, comparing ACM vs. HC cMSC. In both volcano plots, the log2(fold-change) and the -log10(p-value) are represented in x-axis and y-axis, respectively. Red and blue dots denote genes significantly overexpressed or decreased in ACM, respectively ($p < 0.05$), while grey dots genes without difference in expression between the two groups.

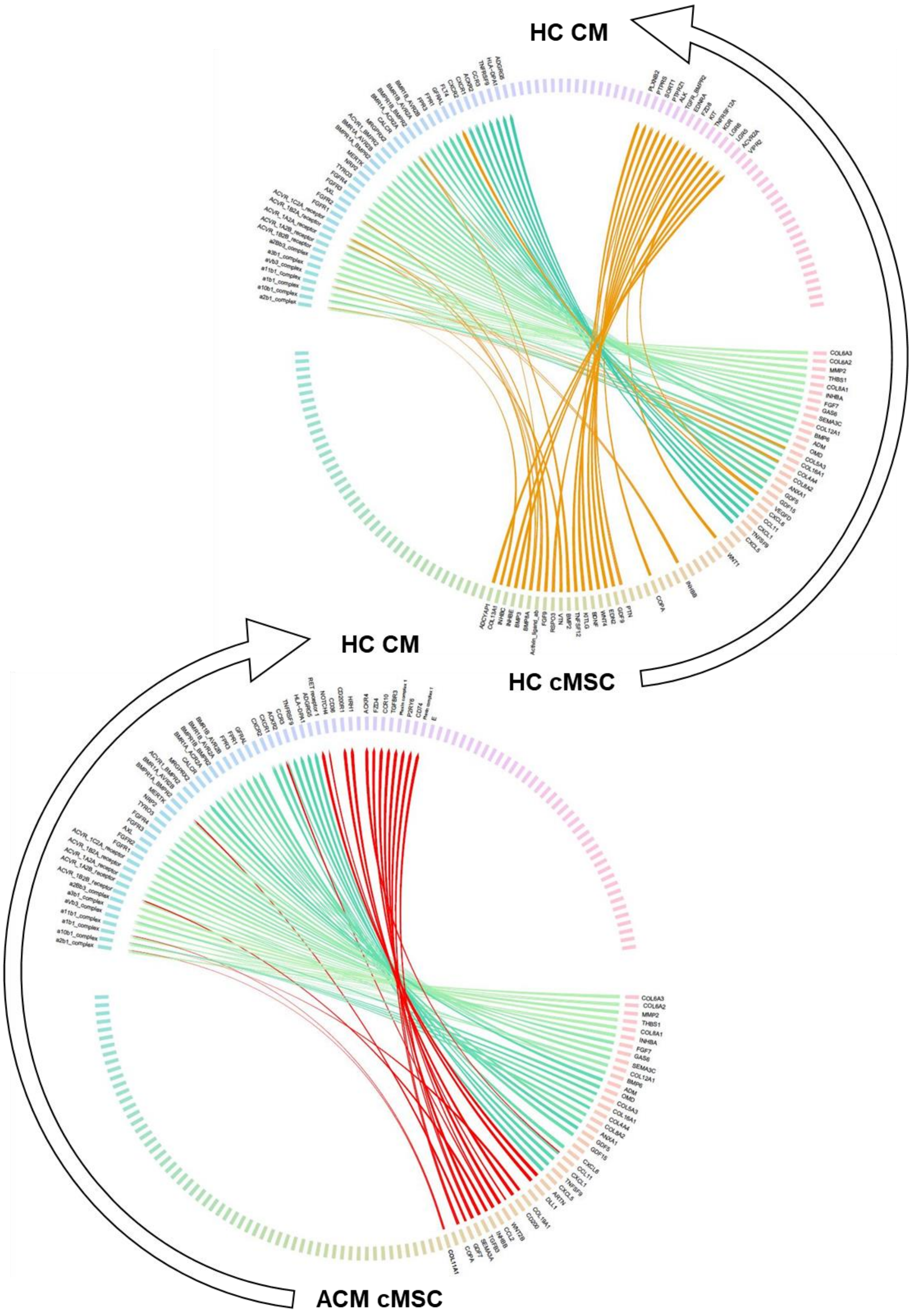

Figure S5. Circle Plot depicting interactions of HC or ACM cMSC → HC CM

Summary of the *in silico*-predicted cell-cell interactions obtained from transcriptomes of homogeneous cultures (n=3 ACM CM cultures, n=3 HC CM and n=3 HC cMSC cultures). The direction of the coloured arrows indicates that the analysis refers to the messages sent from ACM CM (lower semi-circle) to HC cMSC receptors (upper semi-circle). Rectangles depict specific ligands (lower semi-circle) or receptors (upper semi-circle) while each link represents a significant interaction. Green lines are interactions found in common to figures S6, therefore not specific for the genotypes, even if differentially represented, while the red connections are those exclusively identified in ACM CM → HC cMSC. The gradient of green interaction reflects the strength of ligand/receptor interaction from low (light green) to high (dark green) intensity. The open arrow indicates the direction of the interaction. Only predicted interactions with $p<0.001$ and with differential log2 (Fold change score) ACM vs HC between -0.5 and 0.5 are reported. Statistics and quantifications are reported in Supplementary DATA S1.

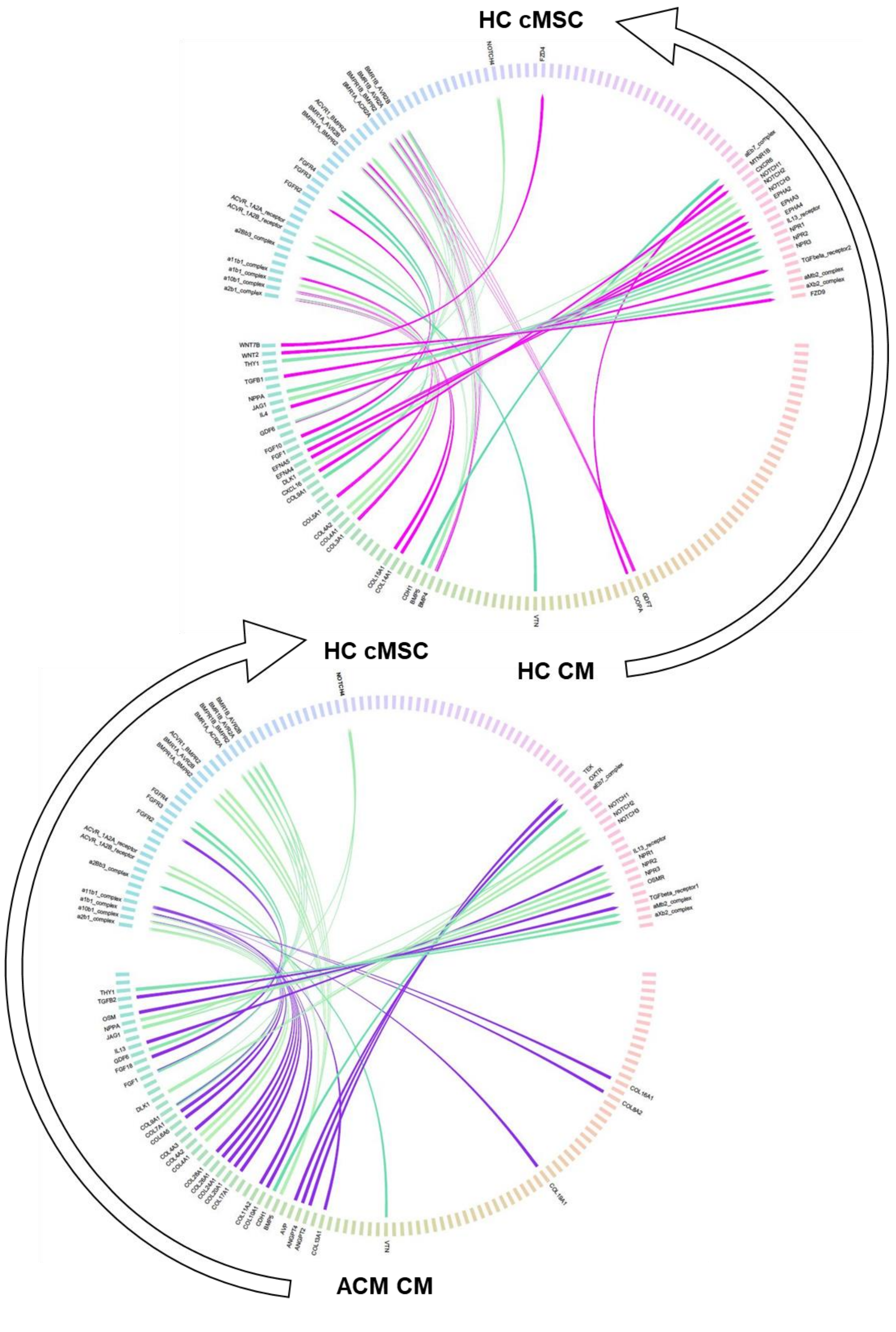

Figure S6. Circle Plot depicting interactions HC or ACM CM → HC cMSC

Summary of the *in silico*-predicted cell-cell interactions obtained from transcriptomes of homogeneous cultures (n=3 HC CM cultures, n=3 ACM CM cultures and n=3 HC cMSC cultures). The direction of the coloured arrows indicates that the analysis refers to the messages sent from HC CM (lower semi-circle of the upper pannel) or ACM CM (lower semi-circle of the upper pannel) to HC cMSC receptors (upper semi-circles). Rectangles depict specific ligands (lower semi-circle) or receptors (upper semi-circle) while each link represents a significant interaction. Green lines are interactions found in common to the upper and lower pannel, therefore not specific for the genotypes, even if differentially represented, while the magenta or purple connections are those exclusively identified in HC CM → HC cMSC (magenta) and ACM CM → HC cMSC (purple). The gradient of green interaction reflects the strength of ligand/receptor interaction from low (light green) to high (dark green) intensity. The open arrow indicates the direction of the interaction. Only predicted interactions with $p<0.001$ and with differential log2 (Fold change score) ACM vs HC between -0.5 and 0.5 are reported. Statistics and quantifications are reported in Supplementary DATA S2.

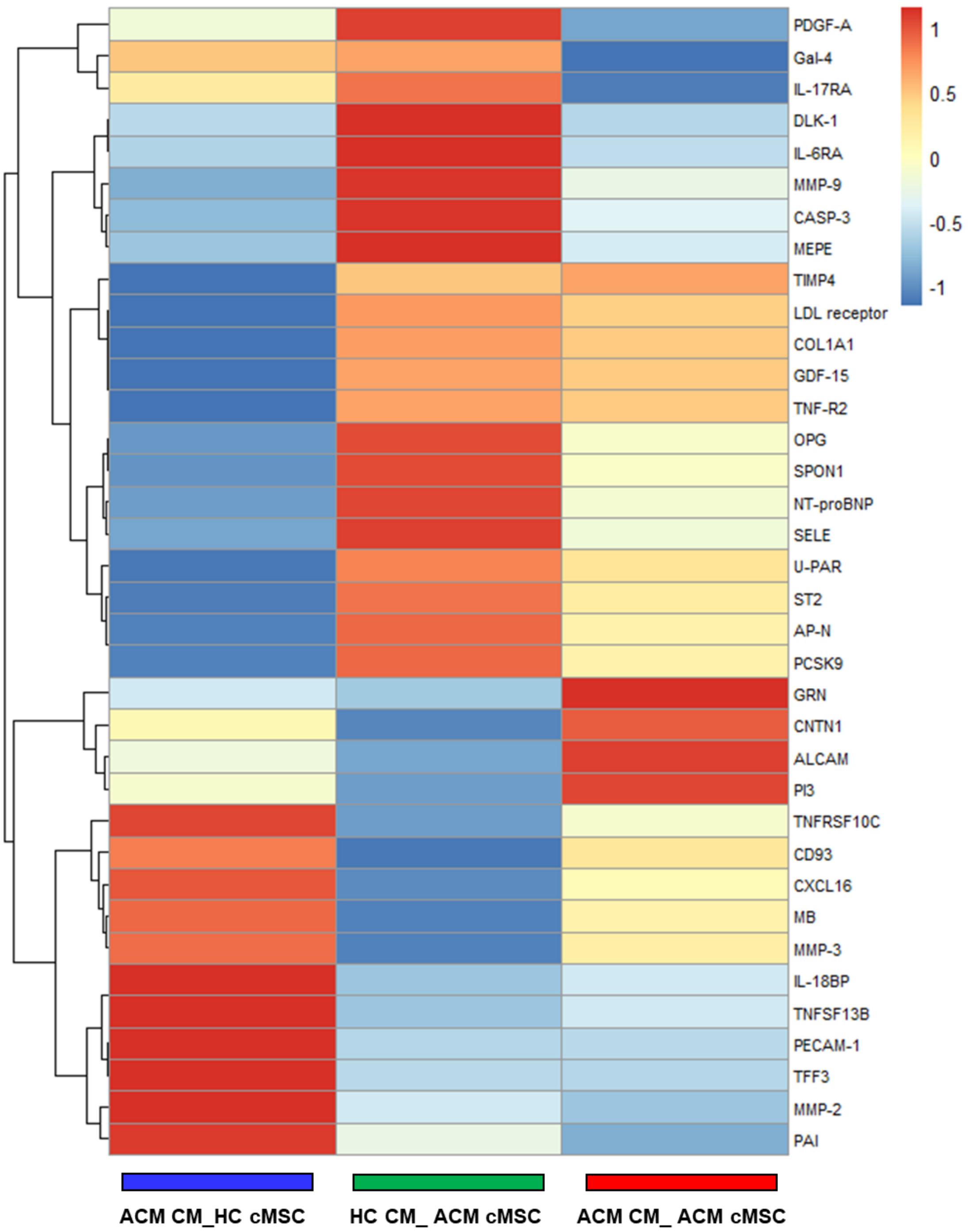

Figure S7. Hit map of differentially secreted factors in the co-cultures

Heatmap visualizing 36 proteins that were found to be significantly (nominative p-value < 0.05) differentially expressed in co-culture conditioned media. The heatmap shows the significant differentially expressed proteins in ACM CM_HC cMSC, HC CM_ACM cMSC and ACM CM_ACM cMSC co-culture media vs. HC CM_HC cMSC co-culture media, whose log of fold change is represented by the colour coding shown in the legend (in red, high expressed proteins; in blue, low-expressed proteins). Rows represent the proteins, whereas the columns represent the specific co-cultures. Co-cultures from 3 independent differentiations were used and statistics are reported in Supplementary DATA S3

Supplementary tables

ACM patient biopsy sample								
**ID**	**Sex/Age (at recruitment)**	**Age/Type of first manifestation**	**Dysfunction/ structural alterations at imaging**	**Tissue characterization**	**Repolarization abnormalities**	**Depolarization conduction abnormalities**	**Arrhythmias**	**Family history/ Genetics**
ACM1	F/ 41	27/PVCs	Major	not conclusive	major	negative	minor	major (*PKP2* c.2013delC p.Lys672ArgfsX12)

Table S1. Summary of bioptic sample collected for Primary stromal cell isolation.

Clinical data of ACM patients enrolled for biopsy samples. Minor and major scores are given according to the International Task Force Criteria for the diagnosis of ACM [1]. VT: ventricular tachycardia; PVCs: premature ventricular contractions. Mutations are reported only when considered pathogenic or likely pathogenic.

iPSC line names	Gender	Age	Ethnicity	Genotype of locus	Disease
LUMCi027-A (https://hpscreg.eu/cell-line/LUMCi027-A)	Female	41	Caucasian	Heterozygous *PKP2* c.2013delC	Arrhythmogenic Cardiomyopathy
LUMCi027-A-1 (https://hpscreg.eu/cell-line/LUMCi027-A-1)	Female	41	Caucasian	*PKP2* WT	Arrhythmogenic Cardiomyopathy (corrected)

Table S2. Summary of hiPSC lines.

Protein	AB	Host	Company	Application/Diluition
**COL1A1**	Monoclonal, #84336	Rabbit	Cell Signaling	IF; 1:200
**α-ACTININ**	Monoclonal, A7811	Mouse	Sigma-Aldrich	IF; 1:800

Table S3. List of antibodies.

References

1 Marcus, F. I. *et al.* Diagnosis of arrhythmogenic right ventricular cardiomyopathy/dysplasia: proposed modification of the task force criteria. *Circulation* **121**, 1533-1541, doi:10.1161/CIRCULATIONAHA.108.840827 (2010).

File Data S1

	reported in the graph (arbitrary choice)
nnnn	interactions specific for HC cMSC ligands
nnnn	common interactions but differentally represented
nnnn	common interactions assumed not differentially represented
nnnn	interactions specific for ACM cMSC ligands

	cMSC --> CM_score				
	**ACM cMSC --> HC CM**	p value	**HC cMSC -> HC CM**	p value	**log2 (Fold change score) ACM vs HC**
PTN & PLXNB2	0.001	p<0,001	243.817	p<0,001	-17.895
PTN & PTPRS	0.001	p<0,001	161.859	p<0,001	-17.304
COPA & SORT1	0.001	p<0,001	140.245	p<0,001	-17.098
PTN & PTPRZ1	0.001	p<0,001	125.279	p<0,001	-16.935
COL5A3 & a11b1 complex	0.001	p<0,001	125.268	p<0,001	-16.935
PTN & ALK	0.001	p<0,001	115.113	p<0,001	-16.813
COL4A4 & a11b1 complex	0.001	p<0,001	113.364	p<0,001	-16.791
GDF9 & TGFR_BMPR2	0.001	p<0,001	93.545	p<0,001	-16.513
EDN2 & EDNRA	0.001	p<0,001	82.191	p<0,001	-16.327
WNT4 & FZD8	0.001	p<0,001	75.844	p<0,001	-16.211
WNT1 & FZD8	0.001	p<0,001	75.026	p<0,001	-16.195
BDNF & SORT1	0.001	p<0,001	74.354	p<0,001	-16.182
KITLG & KIT	0.001	p<0,001	73.269	p<0,001	-16.161
COL5A3 & a2b1 complex	0.001	p<0,001	65.340	p<0,001	-15.996
COL4A4 & a2b1 complex	0.001	p<0,001	53.436	p<0,001	-15.706
TNFSF12 & TNFRSF12A	0.001	p<0,001	52.993	p<0,001	-15.694
BMP2 & BMR1A_ACR2A	0.001	p<0,001	48.627	p<0,001	-15.569
GDF5 & BMR1A_ACR2A	0.001	p<0,001	33.655	p<0,001	-15.039
VTN & aVb3 complex	0.001	p<0,001	33.385	p<0,001	-15.027
VEGFD & KDR	0.001	p<0,001	23.366	p<0,001	-14.512
RSPO3 & LGR6	0.001	p<0,001	17.094	p<0,001	-14.061
RSPO3 & LGR5	0.001	p<0,001	16.841	p<0,001	-14.040
FGF9 & FGFR2	0.001	p<0,001	16.576	p<0,001	-14.017
Activin ligand ab & ACVR_1A2A receptor	0.001	p<0,001	13.494	p<0,001	-13.720
Activin ligand ab & ACVR_1B2A receptor	0.001	p<0,001	13.494	p<0,001	-13.720
INHBB & ACVR_1A2A receptor	0.001	p<0,001	13.494	p<0,001	-13.720
INHBB & ACVR_1B2A receptor	0.001	p<0,001	13.494	p<0,001	-13.720
BMP8A & ACVR_1A2A receptor	0.001	p<0,001	12.801	p<0,001	-13.644
BMP8A & BMR1A_ACR2A	0.001	p<0,001	12.801	p<0,001	-13.644
BMP3 & ACVR_1B2A receptor	0.001	p<0,001	12.689	p<0,001	-13.631
INHBE & ACVR2A	0.001	p<0,001	12.642	p<0,001	-13.626
INHBC & ACVR2A	0.001	p<0,001	12.630	p<0,001	-13.625
COL13A1 & a10b1 complex	0.001	p<0,001	12.355	p<0,001	-13.593
ADCYAP1 & VIPR2	0.001	p<0,001	2.480	p<0,001	-11.277
VEGFD & FLT4	0.743	p<0,001	18.763	p<0,001	-4.657
ADM & CALCR	27.417	p<0,001	79.634	p<0,001	-1.538
ADM & MRGPRX2	27.420	p<0,001	79.637	p<0,001	-1.538
GDF5 & BMPR1B_BMPR2	8.088	p<0,001	22.139	p<0,001	-1.453
GDF5 & BMR1B_AVR2A	8.088	p<0,001	22.139	p<0,001	-1.453
GDF5 & BMR1B_AVR2B	8.088	p<0,001	22.139	p<0,001	-1.453
COL5A3 & a10b1 complex	25.702	p<0,001	60.132	p<0,001	-1.226
COL4A4 & a10b1 complex	23.218	p<0,001	48.228	p<0,001	-1.055
FGF7 & FGFR4	125.930	p<0,001	257.794	p<0,001	-1.034
FGF7 & FGFR3	130.406	p<0,001	262.269	p<0,001	-1.008
FGF7 & FGFR2	141.931	p<0,001	273.794	p<0,001	-0.948
OMD & aVb3 complex	41.326	p<0,001	71.652	p<0,001	-0.794
COL6A2 & a10b1 complex	2876.922	p<0,001	4862.802	p<0,001	-0.757
COL6A2 & a2b1 complex	2882.130	p<0,001	4868.010	p<0,001	-0.756
COL6A2 & a11b1 complex	2942.058	p<0,001	4927.938	p<0,001	-0.744
COL6A2 & a1b1 complex	2955.108	p<0,001	4940.988	p<0,001	-0.742
FGF7 & FGFR1	203.026	p<0,001	334.890	p<0,001	-0.722
MMP2 & aVb3 complex	1824.633	p<0,001	2872.878	p<0,001	-0.655
COL6A3 & a10b1 complex	4084.207	p<0,001	6307.484	p<0,001	-0.627
COL6A3 & a2b1 complex	4089.416	p<0,001	6312.692	p<0,001	-0.626
COL6A3 & a11b1 complex	4149.343	p<0,001	6372.620	p<0,001	-0.619
COL6A3 & a1b1 complex	4162.394	p<0,001	6385.670	p<0,001	-0.617

FAM3C & GPR42	56.270	p<0,001	81.948	p<0,001	-0.542
FAM3C & GLRA2	56.289	p<0,001	81.967	p<0,001	-0.542
COL6A1 & a10b1 complex	3418.350	p<0,001	4925.120	p<0,001	-0.527
COL6A1 & a2b1 complex	3423.558	p<0,001	4930.328	p<0,001	-0.526
COL6A1 & a11b1 complex	3483.486	p<0,001	4990.256	p<0,001	-0.519
COL6A1 & a1b1 complex	3496.536	p<0,001	5003.306	p<0,001	-0.517
C3 & aMb2 complex	124.244	p<0,001	166.368	p<0,001	-0.421
C3 & C3AR1	124.254	p<0,001	166.379	p<0,001	-0.421
COL5A3 & a1b1 complex	103.889	p<0,001	138.318	p<0,001	-0.413
BMP2 & SMO	64.272	p<0,001	81.039	p<0,001	-0.334
HLA-E & CD94:NKG2A	115.864	p<0,001	142.993	p<0,001	-0.304
HLA-E & KLRC1	115.864	p<0,001	142.993	p<0,001	-0.304
CD99 & PILRA	131.252	p<0,001	152.611	p<0,001	-0.218
APP & CD74	554.450	p<0,001	581.205	p<0,001	-0.068
LAMC1 & a7b1 complex	1349.676	p<0,001	1309.507	p<0,001	0.044
LAMC1 & aVb3 complex	1341.978	p<0,001	1301.809	p<0,001	0.044
LAMC1 & a6b1 complex	1320.176	p<0,001	1280.008	p<0,001	0.045
LAMC1 & a2b1 complex	1318.356	p<0,001	1278.187	p<0,001	0.045
FN1 & aVb1 complex	16713.385	p<0,001	14748.172	p<0,001	0.180
FN1 & aVb5 complex	16470.357	p<0,001	14505.145	p<0,001	0.183
FN1 & a11b1 complex	16390.188	p<0,001	14424.976	p<0,001	0.184
FN1 & a3b1 complex	16380.841	p<0,001	14415.629	p<0,001	0.184
FN1 & a5b1 complex	16363.050	p<0,001	14397.838	p<0,001	0.185
FN1 & aVb3 complex	16353.881	p<0,001	14388.669	p<0,001	0.185
FN1 & a2b1 complex	16330.260	p<0,001	14365.048	p<0,001	0.185
FN1 & a10b1 complex	16325.051	p<0,001	14359.839	p<0,001	0.185
FN1 & a8b1 complex	16324.715	p<0,001	14359.503	p<0,001	0.185
FN1 & a4b1 complex	16324.507	p<0,001	14359.295	p<0,001	0.185
FN1 & a2Bb3 complex	16320.942	p<0,001	14355.730	p<0,001	0.185
COL1A2 & a1b1 complex	5420.853	p<0,001	4662.847	p<0,001	0.217
COL1A2 & a11b1 complex	5407.802	p<0,001	4649.797	p<0,001	0.218
COL1A2 & a2b1 complex	5347.875	p<0,001	4589.869	p<0,001	0.221
COL1A2 & a10b1 complex	5342.666	p<0,001	4584.660	p<0,001	0.221
EFNA5 & EPHA7	58.134	p<0,001	49.092	p<0,001	0.244
COL1A1 & a1b1 complex	8870.673	p<0,001	7124.830	p<0,001	0.316
COL1A1 & a11b1 complex	8857.623	p<0,001	7111.780	p<0,001	0.317
COL1A1 & a2b1 complex	8797.695	p<0,001	7051.852	p<0,001	0.319
COL1A1 & a10b1 complex	8792.486	p<0,001	7046.644	p<0,001	0.319
GDF7 & BMR1A_AVR2B	60.271	p<0,001	46.026	p<0,001	0.389
FBN1 & a5b1 complex	1186.390	p<0,001	886.691	p<0,001	0.420
WNT5A & FZD1	303.128	p<0,001	225.684	p<0,001	0.426
COL16A1 & a10b1 complex	75.068	p<0,001	53.496	p<0,001	0.489
BMP6 & BMPR1A_BMPR2	157.385	p<0,001	109.766	p<0,001	0.520
BMP6 & BMR1A_AVR2B	157.385	p<0,001	109.766	p<0,001	0.520
SEMA3C & NRP2	368.343	p<0,001	249.656	p<0,001	0.561
BMP6 & ACVR_1A2B receptor	138.081	p<0,001	90.461	p<0,001	0.610
BMP6 & ACVR1_BMPR2	138.081	p<0,001	90.461	p<0,001	0.610
ANXA1 & FPR3	45.702	p<0,001	29.643	p<0,001	0.625
ANXA1 & FPR1	45.390	p<0,001	29.331	p<0,001	0.630
TNFSF9 & TNFRSF9	9.772	p<0,001	6.203	p<0,001	0.656
COL8A2 & a10b1 complex	61.681	p<0,001	38.994	p<0,001	0.662
TNFSF9 & HLA-DPA1	9.641	p<0,001	6.071	p<0,001	0.667
BMP6 & ACVR_1A2A receptor	126.798	p<0,001	79.178	p<0,001	0.679
BMP6 & BMR1A_ACR2A	126.798	p<0,001	79.178	p<0,001	0.679
TNFSF9 & ADGRG5	9.267	p<0,001	5.698	p<0,001	0.702
GDF15 & GFRAL	34.057	p<0,001	20.844	p<0,001	0.708
BMP6 & BMPR1B_BMPR2	115.282	p<0,001	67.663	p<0,001	0.769
BMP6 & BMR1B_AVR2A	115.282	p<0,001	67.663	p<0,001	0.769
BMP6 & BMR1B_AVR2B	115.282	p<0,001	67.663	p<0,001	0.769
CXCL5 & CXCR2	8.813	p<0,001	4.955	p<0,001	0.831
CCL11 & ACKR2	20.986	p<0,001	11.377	p<0,001	0.883
CCL11 & CCR3	20.427	p<0,001	10.817	p<0,001	0.917
THBS1 & a3b1 complex	5485.530	p<0,001	2756.792	p<0,001	0.993
THBS1 & aVb3 complex	5458.569	p<0,001	2729.832	p<0,001	1.000
THBS1 & a2Bb3 complex	5425.630	p<0,001	2696.893	p<0,001	1.008
CXCL6 & CXCR2	32.162	p<0,001	15.560	p<0,001	1.047
CXCL5 & CXCR1	7.230	p<0,001	3.372	p<0,001	1.100

CXCL6 & CXCR1	30.579	p<0,001	13.977	p<0,001	1.129
INHBA & ACVR_1B2B receptor	972.721	p<0,001	424.451	p<0,001	1.196
INHBA & ACVR_1A2B receptor	949.672	p<0,001	401.401	p<0,001	1.242
INHBA & ACVR_1A2A receptor	938.389	p<0,001	390.118	p<0,001	1.266
INHBA & ACVR_1B2A receptor	938.389	p<0,001	390.118	p<0,001	1.266
INHBA & ACVR_1C2A receptor	925.889	p<0,001	377.618	p<0,001	1.294
GAS6 & AXL	692.078	p<0,001	269.318	p<0,001	1.362
GAS6 & TYRO3	674.378	p<0,001	251.619	p<0,001	1.422
CXCL1 & CXCR2	20.847	p<0,001	7.745	p<0,001	1.428
COL12A1 & a1b1 complex	583.948	p<0,001	215.555	p<0,001	1.438
GAS6 & MERTK	661.268	p<0,001	238.509	p<0,001	1.471
COL12A1 & a11b1 complex	570.898	p<0,001	202.505	p<0,001	1.495
CXCL1 & CXCR1	19.265	p<0,001	6.163	p<0,001	1.644
COL12A1 & a2b1 complex	510.970	p<0,001	142.578	p<0,001	1.841
COL12A1 & a10b1 complex	505.762	p<0,001	137.369	p<0,001	1.880
COL8A1 & a1b1 complex	1925.337	p<0,001	514.992	p<0,001	1.902
COL8A1 & a11b1 complex	1912.287	p<0,001	501.942	p<0,001	1.930
COL8A1 & a2b1 complex	1852.359	p<0,001	442.014	p<0,001	2.067
COL8A1 & a10b1 complex	1847.151	p<0,001	436.805	p<0,001	2.080
ARTN & RET receptor 1	1.461	p<0,001	0.001	p<0,001	10.514
DLL1 & NOTCH4	2.008	p<0,001	0.001	p<0,001	10.972
WNT1 & CD36	3.402	p<0,001	0.001	p<0,001	11.733
COL19A1 & a10b1 complex	7.694	p<0,001	0.001	p<0,001	12.910
CD200 & CD200R1	14.894	p<0,001	0.001	p<0,001	13.863
IL6 & HRH1	21.109	p<0,001	0.001	p<0,001	14.366
IL6 & IL6 receptor	21.990	p<0,001	0.001	p<0,001	14.425
CCL11 & ACKR4	23.481	p<0,001	0.001	p<0,001	14.519
WNT2B & FZD4	29.158	p<0,001	0.001	p<0,001	14.832
CCL2 & ACKR2	42.998	p<0,001	0.001	p<0,001	15.392
CCL2 & CCR10	44.738	p<0,001	0.001	p<0,001	15.449
INHBB & ACVR_1B2B receptor	47.017	p<0,001	0.001	p<0,001	15.521
TGFB3 & TGFBR3	49.395	p<0,001	0.001	p<0,001	15.592
SEMA3A & PlexinA2_complex1	51.290	p<0,001	0.001	p<0,001	15.646
GDF7 & BMPR1A_BMPR2	60.271	p<0,001	0.001	p<0,001	15.879
COPA & P2RY6	87.019	p<0,001	0.001	p<0,001	16.409
COPA & CD74	87.693	p<0,001	0.001	p<0,001	16.420
SEMA3A & PlexinA4_complex1	97.398	p<0,001	0.001	p<0,001	16.572
WNT5A & EPHA7	151.578	p<0,001	0.001	p<0,001	17.210
WNT5A & ROR2	157.366	p<0,001	0.001	p<0,001	17.264
COL11A1 & a10b1 complex	162.740	p<0,001	0.001	p<0,001	17.312
COL11A1 & a2b1 complex	167.949	p<0,001	0.001	p<0,001	17.358
VEGFB & NRP1	183.957	p<0,001	0.001	p<0,001	17.489
COL11A1 & a1b1 complex	240.927	p<0,001	0.001	p<0,001	17.878

File Data S2

	reported in the graph (arbitrary choice)
nnnn	interactions specific for HC cMSC ligands
nnnn	common interactions but differentally represented
nnnn	common interactions assumed not differentially represented
nnnn	interactions specific for ACM cMSC ligands

CM --> CMSC_score

	**ACM CM --> HC cMSC**	p value	**HC CM --> HC cMSC**	p value	**log2 (Fold change score) ACM vs HC**
COL3A1 & a11b1 complex	0.001	p<0,001	4536.622	p<0,001	-22.113
COL3A1 & a2b1 complex	0.001	p<0,001	4490.446	p<0,001	-22.098
COL3A1 & a10b1 complex	0.001	p<0,001	4460.438	p<0,001	-22.089
COL5A1 & a11b1 complex	0.001	p<0,001	1150.412	p<0,001	-20.134
COL5A1 & a2b1 complex	0.001	p<0,001	1104.236	p<0,001	-20.075
COL5A1 & a10b1 complex	0.001	p<0,001	1074.227	p<0,001	-20.035
VEGFB & NRP1	0.001	p<0,001	208.622	p<0,001	-17.671
GDF6 & BMR1A_AVR2B	0.001	p<0,001	160.416	p<0,001	-17.291
COL14A1 & a10b1 complex	0.001	p<0,001	143.249	p<0,001	-17.128
WNT5A & ROR2	0.001	p<0,001	136.825	p<0,001	-17.062
WNT5A & EPHA7	0.001	p<0,001	117.043	p<0,001	-16.837
BMP4 & BMPR1A_BMPR2	0.001	p<0,001	104.135	p<0,001	-16.668
COL15A1 & a10b1 complex	0.001	p<0,001	89.671	p<0,001	-16.452
BMP4 & BMR1A_ACR2A	0.001	p<0,001	85.449	p<0,001	-16.383
TGFB1 & TGFbeta receptor2	0.001	p<0,001	81.500	p<0,001	-16.315
BMP4 & BMR1A_AVR2B	0.001	p<0,001	80.257	p<0,001	-16.292
COPA & MTNR1B	0.001	p<0,001	78.528	p<0,001	-16.261
BMP4 & BMPR1B_BMPR2	0.001	p<0,001	77.540	p<0,001	-16.243
BMP4 & BMR1B_AVR2A	0.001	p<0,001	77.540	p<0,001	-16.243
BMP4 & BMR1B_AVR2B	0.001	p<0,001	77.540	p<0,001	-16.243
WNT7B & FZD4	0.001	p<0,001	65.102	p<0,001	-15.990
COL11A1 & a10b1 complex	0.001	p<0,001	55.559	p<0,001	-15.762
EFNA5 & EPHA4	0.001	p<0,001	35.748	p<0,001	-15.126
EFNA5 & EPHA3	0.001	p<0,001	34.866	p<0,001	-15.090
FGF10 & FGFR2	0.001	p<0,001	26.859	p<0,001	-14.713
EFNA4 & EPHA2	0.001	p<0,001	25.956	p<0,001	-14.664
IL4 & IL13 receptor	0.001	p<0,001	25.761	p<0,001	-14.653
GDF7 & BMR1A_ACR2A	0.001	p<0,001	25.607	p<0,001	-14.644
GDF7 & BMPR1B_BMPR2	0.001	p<0,001	17.698	p<0,001	-14.111
GDF7 & BMR1B_AVR2A	0.001	p<0,001	17.698	p<0,001	-14.111
GDF7 & BMR1B_AVR2B	0.001	p<0,001	17.698	p<0,001	-14.111
WNT1 & CD36	0.001	p<0,001	13.385	p<0,001	-13.708
WNT2 & FZD9	0.001	p<0,001	11.116	p<0,001	-13.440
CXCL16 & CXCR6	0.001	p<0,001	5.362	p<0,001	-12.389
DLK1 & NOTCH4	149.069	p<0,001	934.626	p<0,001	-2.648
DLK1 & NOTCH1	172.008	p<0,001	957.565	p<0,001	-2.477
DLK1 & NOTCH3	190.446	p<0,001	976.003	p<0,001	-2.357
CDH1 & aEb7 complex	2.074	p<0,001	9.728	p<0,001	-2.229
DLK1 & NOTCH2	326.707	p<0,001	1112.264	p<0,001	-1.767
THY1 & aXb2 complex	27.076	p<0,001	79.402	p<0,001	-1.552
THY1 & aMb2 complex	27.114	p<0,001	79.439	p<0,001	-1.551
VTN & a2Bb3 complex	2.917	p<0,001	8.297	p<0,001	-1.508
GDF6 & BMPR1B_BMPR2	74.511	p<0,001	157.698	p<0,001	-1.082
GDF6 & BMR1B_AVR2A	74.511	p<0,001	157.698	p<0,001	-1.082
GDF6 & BMR1B_AVR2B	74.511	p<0,001	157.698	p<0,001	-1.082
COL4A1 & a10b1 complex	680.331	p<0,001	1381.644	p<0,001	-1.022
GDF6 & BMR1A_ACR2A	82.421	p<0,001	165.608	p<0,001	-1.007
COL4A1 & a1b1 complex	699.203	p<0,001	1400.516	p<0,001	-1.002
COL4A1 & a2b1 complex	710.340	p<0,001	1411.653	p<0,001	-0.991
JAG1 & NOTCH4	116.928	p<0,001	192.730	p<0,001	-0.721
COL4A2 & a10b1 complex	786.667	p<0,001	1229.805	p<0,001	-0.645
COL4A2 & a1b1 complex	805.539	p<0,001	1248.677	p<0,001	-0.632
COL4A2 & a2b1 complex	816.676	p<0,001	1259.814	p<0,001	-0.625
JAG1 & NOTCH1	139.867	p<0,001	215.669	p<0,001	-0.625
COL4A2 & a11b1 complex	862.853	p<0,001	1305.991	p<0,001	-0.598

WNT5A & FZD3	78.897	p<0,001	117.696	p<0,001	-0.577
WNT5A & FZD5	80.258	p<0,001	119.057	p<0,001	-0.569
JAG1 & NOTCH3	158.305	p<0,001	234.107	p<0,001	-0.564
TGFB1 & TGFBR3	72.484	p<0,001	95.987	p<0,001	-0.405
WNT5A & ROR1	122.942	p<0,001	161.741	p<0,001	-0.396
EFNB1 & EPHB4	78.621	p<0,001	102.972	p<0,001	-0.389
CXCL12 & DPP4	447.282	p<0,001	542.825	p<0,001	-0.279
WNT5A & FZD2	182.900	p<0,001	221.699	p<0,001	-0.278
AGT & AGTR1	31.199	p<0,001	36.707	p<0,001	-0.235
WNT2 & FZD4	65.539	p<0,001	76.012	p<0,001	-0.214
VEGFA & GRIN2B	174.698	p<0,001	202.183	p<0,001	-0.211
VEGFA & FLT1	175.443	p<0,001	202.927	p<0,001	-0.210
VEGFA & FLT1 complex	175.443	p<0,001	202.927	p<0,001	-0.210
VEGFA & KDR	179.746	p<0,001	207.231	p<0,001	-0.205
VEGFA & EPHB2	181.329	p<0,001	208.813	p<0,001	-0.204
WNT2 & FZD2	105.218	p<0,001	115.690	p<0,001	-0.137
VEGFA & NRP1	351.324	p<0,001	378.809	p<0,001	-0.109
FGF18 & FGFR4	17.512	p<0,001	13.153	p<0,001	0.413
FGF18 & FGFR3	17.490	p<0,001	13.131	p<0,001	0.414
NPPA & NPR3	200.996	p<0,001	140.464	p<0,001	0.517
FGF1 & FGFR4	22.629	p<0,001	15.305	p<0,001	0.564
FGF1 & FGFR3	22.606	p<0,001	15.282	p<0,001	0.565
COL9A1 & a2b1 complex	78.501	p<0,001	50.355	p<0,001	0.641
BMP5 & ACVR1_BMPR2	339.428	p<0,001	208.969	p<0,001	0.700
BMP5 & BMPR1A_BMPR2	334.433	p<0,001	203.974	p<0,001	0.713
BMP5 & ACVR_1A2A receptor	315.748	p<0,001	185.288	p<0,001	0.769
BMP5 & BMR1A_ACR2A	315.748	p<0,001	185.288	p<0,001	0.769
BMP5 & ACVR_1A2B receptor	310.556	p<0,001	180.096	p<0,001	0.786
BMP5 & BMR1A_AVR2B	310.556	p<0,001	180.096	p<0,001	0.786
BMP5 & BMPR1B_BMPR2	307.838	p<0,001	177.379	p<0,001	0.795
BMP5 & BMR1B_AVR2A	307.838	p<0,001	177.379	p<0,001	0.795
BMP5 & BMR1B_AVR2B	307.838	p<0,001	177.379	p<0,001	0.795
NPPA & NPR2	127.029	p<0,001	66.497	p<0,001	0.934
NPPA & NPR1	110.592	p<0,001	50.059	p<0,001	1.144
COL9A1 & a10b1 complex	48.492	p<0,001	20.346	p<0,001	1.253
IL6 & IL6 receptor	9.901	p<0,001	0.001	p<0,001	13.274
ANGPT4 & TEK	11.024	p<0,001	0.001	p<0,001	13.428
ANGPT2 & TEK	12.405	p<0,001	0.001	p<0,001	13.599
COL7A1 & a10b1 complex	15.379	p<0,001	0.001	p<0,001	13.909
FGF18 & FGFR2	17.469	p<0,001	0.001	p<0,001	14.093
AVP & OXTR	19.012	p<0,001	0.001	p<0,001	14.215
FGF1 & FGFR2	22.586	p<0,001	0.001	p<0,001	14.463
IL13 & IL13 receptor	25.767	p<0,001	0.001	p<0,001	14.653
OSM & OSMR	62.488	p<0,001	0.001	p<0,001	15.931
COL9A1 & a1b1 complex	67.364	p<0,001	0.001	p<0,001	16.040
COL20A1 & a11b1 complex	78.946	p<0,001	0.001	p<0,001	16.269
COL6A5 & a11b1 complex	78.946	p<0,001	0.001	p<0,001	16.269
COL24A1 & a11b1 complex	79.073	p<0,001	0.001	p<0,001	16.271
COL10A1 & a11b1 complex	79.103	p<0,001	0.001	p<0,001	16.271
COL28A1 & a11b1 complex	79.170	p<0,001	0.001	p<0,001	16.273
COL17A1 & a11b1 complex	79.364	p<0,001	0.001	p<0,001	16.276
COL4A3 & a11b1 complex	79.622	p<0,001	0.001	p<0,001	16.281
COL26A1 & a11b1 complex	79.958	p<0,001	0.001	p<0,001	16.287
COL8A2 & a11b1 complex	81.116	p<0,001	0.001	p<0,001	16.308
COL19A1 & a11b1 complex	82.057	p<0,001	0.001	p<0,001	16.324
COL13A1 & a11b1 complex	82.734	p<0,001	0.001	p<0,001	16.336
COL11A2 & a1b1 complex	83.717	p<0,001	0.001	p<0,001	16.353
COL7A1 & a11b1 complex	91.564	p<0,001	0.001	p<0,001	16.483
COL16A1 & a11b1 complex	95.432	p<0,001	0.001	p<0,001	16.542
TGFB2 & TGFbeta receptor1	97.387	p<0,001	0.001	p<0,001	16.571

File Data S3

reported in the graph (Figure S6)

	NPX mean				
	HC CM_HC cMSC	ACM CM_HC cMSC	ACM cMSC_HC CM	ACM CM _ACM cMSC	**p value**
ALCAM	316.8	313	271.4	387.3	p<0,05
AP-N	116.9	95.83	194.3	157	p<0,01
AXL	784	712.9	701.5	724.8	
AZU1	0.8047	0.9059	0.7819	0.869	
BLM hydrolase	71.32	58.7	68.94	62.15	
CASP-3	1850	1237	1781	1353	p<0,01
CCL15	1.139	1.133	1.134	1.19	
CCL16	0.9537	0.8963	0.8934	0.9136	
CCL24	1.47	1.52	1.567	1.599	
CD163	1.412	1.499	1.481	1.41	
CD93	58.35	48.18	32.34	43.8	p<0,01
CDH5	1.448	1.339	1.221	1.231	
CHI3L1	357.6	369.2	345.1	330.2	
CHIT1	0.3252	0.2842	0.2253	0.2468	
CNTN1	1.148	1.331	1.18	1.446	p<0,0001
COL1A1	7.949	9.02	11.67	11.36	p<0,01
CPA1	17.24	11.05	14.9	12.67	
CSTB	696.5	683.6	750.3	749.7	
CTSD	160.8	163.6	142.3	151.5	
CTSZ	215.8	212.6	206.4	203.8	
CXCL16	99.95	95.69	63.95	81.03	p<0,01
DLK-1	1103	59.72	1406	49.51	p<0,0001
EGFR	7.162	6.514	6.325	7.702	
Ep-CAM	24.67	29.64	24.91	30.75	
EPHB4	60.96	56.36	76.53	72.19	
FAS	3218	1733	6446	4036	
Gal-3	293.1	317.1	323.7	352	
Gal-4	1.192	1.18	1.198	1.012	p<0,05
GDF-15	2668	1069	4406	4063	p<0,01
GP6	0.9505	0.9041	0.8967	0.8895	
GRN	903.6	794.9	790.7	824.1	p<0,05
ICAM-2	1.416	1.437	1.29	1.31	
IL-17RA	29.28	22.55	23.87	19.64	p<0,01
IL-18BP	53.76	42.92	37.35	38.1	p<0,01
IL-1RT1	125.7	96.45	130.9	166.7	
IL-1RT2	2.452	2.276	2.566	2.411	
IL2-RA	1.169	1.192	1.19	1.196	
IL-6RA	217.8	121.9	211.4	124.6	p<0,0001
ITGB2	1.153	1.125	1.165	1.059	
JAM-A	109.7	103.3	98.75	106.6	
KLK6	1.707	1.742	1.718	2.105	
LDL receptor	710.4	582.2	874.6	833.7	p<0,0001
LTBR	91.05	74.91	111.6	113.3	
MB	677.6	1369	583.3	1063	p<0,0001
MCP-1	15250	17681	16616	17797	
MEPE	28.55	10.92	31.62	14.1	p<0,01
MMP-2	396	404.5	358	350.6	p<0,01
MMP-3	3788	3247	2784	3083	p<0,05
MMP-9	2.273	2.426	6.154	3.498	p<0,05
MPO	0.8436	0.9474	0.8847	0.892	
Notch 3	51.6	38.67	62.02	61.83	
NT-proBNP	15473	11163	14479	12489	p<0,0001
OPG	5612	4366	5628	4925	p<0,01
OPN	3063	3419	2697	3228	
PAI	285.5	305.5	275.2	262.4	p<0,01
PCSK9	11.61	9.673	13.71	12.17	p<0,01
PDGF-A	113.9	89.51	103.8	81.35	p<0,01
PECAM-1	6.108	5.76	2.69	2.733	p<0,0001
PGLYRP1	0.8362	0.9528	0.8538	0.8411	
PI3	265	329.1	307.1	359.1	p<0,01
PLC	926.5	918.4	928.3	898.8	
PON3	1.059	1.007	1.01	1	

PSP-D	0.8939	0.9559	0.956	1.001	
RARRES2	2670	2671	1685	2812	
RETN	0.8503	0.9977	1.109	1.169	
SCGB3A2	0.9838	0.8814	0.8652	0.8299	
SELE	114.4	143.9	265	187.6	p<0,01
SELP	3.148	3.703	3.952	3.668	
SHPS-1	45.94	39.66	49.47	44.18	
SPON1	2.874	1.993	3.238	2.594	p<0,01
ST2	12.86	8.626	13.44	11.81	p<0,01
TFF3	1.166	1.37	1.205	1.204	p<0,05
TFPI	2230	1828	2851	2818	
TIMP4	43.18	32.38	55.96	58.26	p<0,01
TLT-2	1.217	1.18	1.274	1.199	
TNF-R1	881.6	726.4	951.2	887.6	
TNF-R2	2.415	2.49	4.723	4.495	p<0,0001
TNFRSF10C	215.2	188.7	159.1	171.6	p<0,01
TNFRSF14	19.94	19.52	15.45	16.41	
TNFSF13B	39.89	37.97	14.61	17.65	p<0,0001
t-PA	255.3	272.5	258.4	247.8	
TR	5.059	3.822	5.299	4.866	
TR-AP	4.557	5.29	7.585	7.154	
uPA	2399	2169	1508	1816	
U-PAR	1487	940.2	2114	1813	p<0,01
Vwf	1.406	1.241	1.321	1.252	
